# ZSH‐2208: A novel retinoid with potent anti‐tumour effects on ESCC stem cells via RARγ–TNFAIP3 axis

**DOI:** 10.1002/ctm2.70148

**Published:** 2024-12-26

**Authors:** Ruoxue Chen, Xuan Huang, Jiayun Hou, Junjie Ni, Wenrui Zhao, Quanlin Li, Heng Jiao, Xin Cao

**Affiliations:** ^1^ Institute of Clinical Science, Zhongshan Hospital Fudan University Shanghai Medical College Shanghai China; ^2^ Endoscopy Center and Endoscopy Research Institute, Zhongshan Hospital Fudan University Shanghai China; ^3^ Shanghai Collaborative Innovation Center of Endoscopy Shanghai China; ^4^ Department of Thoracic Surgery, Zhongshan Hospital Fudan University Shanghai Medical College Shanghai China

**Keywords:** oesophageal squamous cell carcinoma, retinoid, retinoid acid receptor γ, TNFAIP3, tumour‐repopulating cells, ZSH‐2208

## Abstract

**Backgroud:**

Oesophageal cancer ranks among the most prevalent malignant tumours globally, primarily consisting of oesophageal squamous cell carcinoma (ESCC). Cancer stem cells (CSCs) accelerate the progression ESCC via their strong self‐renewal and tumourigenic capabilities, presenting significant clinical challenges due to increased risks of recurrence and drug resistance.

**Methods:**

Our previous study has reported WYC‐209, which is capable of inducing apoptosis of CSCs in melanoma and hepatoma, but is ineffective against ESCC. Additionally, clinical studies in ESCC still lack drug candidates that effectively target CSCs. Therefore, our team developed a series of novel retinoids that target retinoic acid receptors (RARs), with enhanced potency, broader efficacy and minimised toxic side effects against CSCs. Following iterative optimisation and pharmacological validation, ZSH‐2208 was identified as the most promising candidate for effectively targeting ESCC tumour‐repopulating cells (TRCs). Mechanistic exploration revealed that ZSH‐2208 inhibits the growth of ESCC‐TRCs through modulation of the RARγ–TNFAIP3 axis. The clinical significance of the key molecule TNFAIP3 in ESCC has also been demonstrated.

**Results:**

This study introduces ZSH‐2208, a novel retinoid specifically targeting ESCC‐TRCs, which holds significant potential for clinical application in ESCC.

**Key points:**

- The ESCC‐TRCs replicates the characteristics of ESCC stem cells, which are inhibited by ZSH‐2208.
- In vivo and in vitro experiments demonstrated that ZSH‐2208, a novel RA analogue, effectively inhibits the growth of ESCC‐TRCs through the RARγ–TNFAIP3 axis.
- Low levels of TNFIP3 protein may be associated with improved survival probability in ESCC patients.

## Graphical abstract

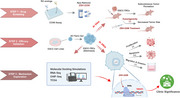

## 1 INTRODUCTION

Oesophageal cancer (EC) is a prevalent malignancy in China, with over 84% of cases identified as oesophageal squamous cell carcinoma (ESCC).^1^ Currently, the standard first‐line treatment for ESCC involves the combination of platinum agents with either fluorouracil or paclitaxel.^2^ However, these treatment options are restricted to a specific subset of patients and are often associated with drug resistance and relapse.

Cancer stem cells (CSCs), known for their resistance to conventional cancer therapies, pose a significant challenge in combating cancer recurrence. Additionally, CSCs have been implicated in the progression, drug resistance and recurrence of ESCC.^3^ Therefore, elucidating the complex mechanisms through which CSCs contribute to tumour resistance and recurrence has emerged as a critical objective in the clinical management of ESCC.^4^ Tumour‐repopulating cells (TRCs), commonly utilised in tumour stemness research, represent CSCs with reduced differentiation potential and enhanced self‐renewal and tumourigenic capacities. Previous studies have confirmed the unique characteristics of TRCs, especially their resistance to chemotherapy drugs.^5^ Targeting TRCs is a novel and promising strategy to combat drug resistance and recurrence in anti‐tumour research.^6^

Retinoic acid (RA), serving as an endogenous ligand for the retinoic acid receptor (RAR) and retinoid X receptor (RXR) family, regulates translational events and cellular adaptability.^7^, ^8^ RARs (RARα, RARβ, RARγ), through homodimerisation or heterodimerisation with RXRs (RXRα, RXRβ, RXRγ), precisely control the transcriptional expression of stemness genes, thereby impacting essential cellular processes, including growth, differentiation, apoptosis and programmed cell death.^9^ All‐trans retinoic acid (ATRA) is the natural and physiological form of RA that exhibits pan‐agonistic activity towards all three types of RA receptors, making it widely utilised in clinical practice. The combination of ATRA with other therapeutic agents, such as chemotherapy, epigenetic modifiers and arsenic trioxide, has been rigorously investigated across various cancer types. ATRA has been demonstrated the capability to reduce the growth and dissemination of human ESCC cell lines. In animal models, ATRA inhibits the progression of EC1 cell xenografts by blocking the angiopoietin‐Tie2 pathway in ESCC tumours.^10^ Moreover, the relationship between RARs and ESCC has been documented in both clinical and experimental studies. For example, the loss of RARβ expression may be associated with the occurrence and progression of ESCC, whereas the up‐regulation of RARβ by RA can inhibit growth and induce apoptosis in ESCC cell lines. Additionally, hypermethylated RARβ plays a crucial role in early‐stage ESCC.^11^

Our previous studies have demonstrated the high susceptibility of TRCs to retinoids. Based on the structural characteristics of RARs, our previous study has developed a targeted RA analogue, WYC‐209, which effectively inhibits the growth of TRCs from various cancers (including melanoma, breast cancer [BC], ovarian cancer and lung cancer) in vitro. Additionally, it suppresses the growth and metastasis of malignant melanoma and hepatocellular carcinoma (HCC) TRCs in vivo, without causing significant harmful side effects.^12^ In this study, a wide range of tumour TRCs were extensively screened to evaluate the growth‐inhibitory effects of these retinoids. Among them, ZSH‐2208 demonstrated a stronger growth‐inhibitory effect on varieties of tumour cells, particularly on ESCC‐TRCs. In vitro, ZSH‐2208 significantly reduced proliferation, migration and invasion while increasing apoptosis in ESCC‐TRCs. In vivo, ZSH‐2208 effectively inhibited tumour growth in nude mouse subcutaneous models without significant toxicity. Further mechanistic studies revealed that ZSH‐2208 down‐regulated RARγ protein expression, which led to decreased transcriptional expression of TNFAIP3 and subsequent growth inhibition in ESCC‐TRCs. Moreover, the analysis of clinical samples indicated that high TNFAIP3 protein expression was correlated with poor prognosis among ESCC patients. Our findings demonstrate that ZSH‐2208 inhibits the proliferation of ESCC‐TRCs via suppressing the transcription of TNFAIP3. Consequently, ZSH‐2208, a novel retinoid, shows promise as a potential candidate for clinical therapy of ESCC.

## 2 RESULTS

### 2.1 The identification of ZSH‐2208

**FIGURE 1:**
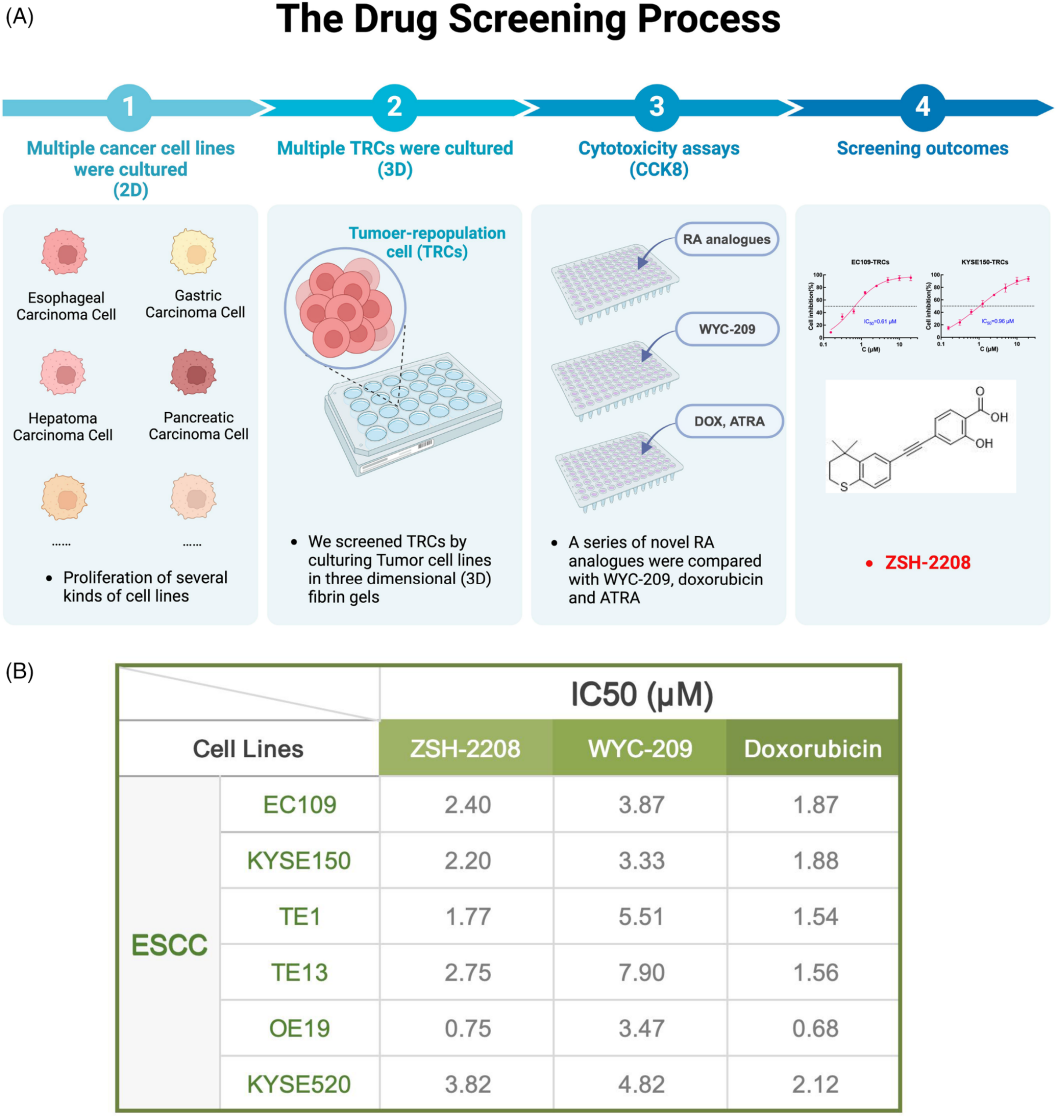
The identification of ZSH‐2208. (A) Process of drug screening and chemical structure of ZSH‐2208. (B) IC_50_ values of ZSH‐2208 against several types of ESCC cell lines.

**FIGURE 2:**
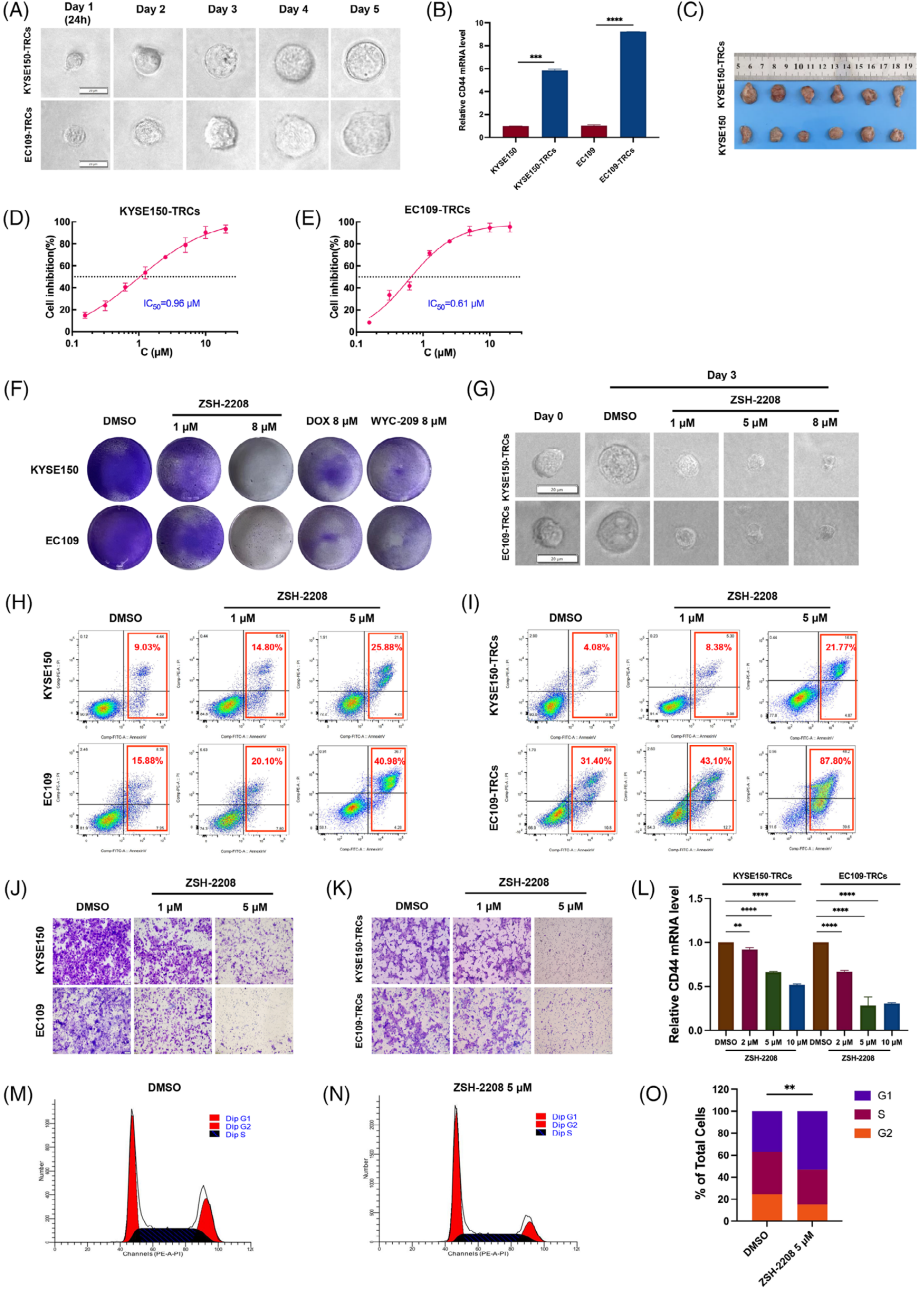
ZSH‐2208 inhibits the growth of ESCC‐TRCs in vitro. (A) The diameter of monoclonal spheres of ESCC‐TRCs gradually increased over time. (B) Expression of CD44 in ESCC‐TRCs detected vis qRT‐PCR. (C) Subcutaneous tumourigenicity of ESCC cells and ESCC‐TRCs in nude mice was compared. (D and E) IC_50_ values of ZSH‐2208 on KYSE150‐TRCs and EC109‐TRCs. (F and G) ZSH‐2208 significantly inhibits the growth of ESCC‐TRCs. DOX, doxorubicin. (H and I) Effect of ZSH‐2208 on apoptosis of ESCC cells. (J and K) Effect of ZSH‐2208 on invasion of ESCC cells and ESCC‐TRCs. (L) ZSH‐2208 significantly decreased the expression of stemness marker CD44 in ESCC‐TRCs. (M, N and O) ZSH‐2208 arrested the cycle of EC109‐TRC in G1 phase. Data are presented as the mean ± SD; *p < .05, **p < .01, ***p < .001, ****p < .0001; ns, not significant.

Our group previously introduced WYC‐209, a novel RA analogue that effectively inhibits tumour growth in vitro and in vivo without causing toxicity.^13^ To enhance the anti‐cancer efficacy of RA analogues, we optimised the structure of WYC‐209 and conducted preliminary screening of these new synthesised RA analogues to investigate their inhibitory effects on tumour cell growth. The tumour cell growth inhibition tests were conducted on various human‐derived tumour cell lines, including those from ESCC, gastric cancer (GC), HCC, pancreatic cancer and colorectal cancer (CRC), along with their respective TRCs (Figure 1A). Compound ZSH‐2208 exhibited potent anti‐tumour effects, surpassing those of WYC‐209 across a variety of tumour cells (Figure 1B). Furthermore, the half inhibitory concentration (IC_50_) of ZSH‐2208 against these tumour cells was meticulously determined and compared with WYC‐209, doxorubicin (DOX) and ATRA. The IC_50_ of ZSH‐2208, WYC‐209, DOX and ATRA against KYSE150, KYSE150‐TRCs, EC109 and EC109‐TRCs were as follows: (1) ZSH‐2208: 2.20, 0.61, 2.40 and 0.75 µM; (2) WYC‐209: 3.33, 1.01, 3.87 and 3.74 µM; (3) DOX: 1.88, 3.72, 1.87 and 2.90 µM; (4) ATRA: 45.32, 49.15, 36.98 and 50.37 µM  2D,E S2B,C,D,E).(Figures 2D,E and S1B,C,D,E2).

The results showed that ZSH‐2208 was more effective than WYC‐209 in inhibiting the growth of tumour cells and was comparable to DOX in multiple cell lines and TRCs. Notably, ZSH‐2208 exhibited superior activity over WYC‐209 specifically in ESCC cells and TRCs. Based on these findings and our drug screening efforts, we have identified ZSH‐2208 as the primary focus of our drug research. The main objective of our study was to investigate and elucidate the inhibitory effects of ZSH‐2208 on ESCC cell growth, providing new research opportunities for the clinical application of novel RA analogues in ESCC treatment.

### 2.2 ZSH‐2208 inhibits the growth of ESCC‐TRCs in vitro and in vivo

CSC like cells, which are TRCs, were systematically developed and validated for their characteristics of stem cells. The clonesphere formation and cell migration assays demonstrated that ESCC‐TRCs exhibited enhanced proliferation and migration abilities compared with ESCC cell lines (Figures 2A and S2A,B). Flow cytometry and qRT‐PCR assays confirmed the expression of CD44 and CR‐1, classical stemness markers of ESCC in ESCC‐TRCs (Figures 2B and S2C,D,E). Additionally, subcutaneous tumourigenicity assay in nude mice revealed a significantly higher tumourigenic potential of ESCC‐TRCs compared with ESCC cell lines (Figures 2C and S2F,G). The stemness of ESCC‐TRCs cultivated in a 3D soft fibrin gel environment is validated through comprehensive in vitro and in vivo experiments, confirming their role as designated ESCC stem cells for further investigations.

The IC_50_ of ZSH‐2208 was determined using a CCK‐8 assay on both ESCC cells and ESCC‐TRCs. 0.1% DMSO served as the negative control (NC), while WYC‐209, DOX and ATRA as positive controls (Figures 1A and S1B,C,D,E). Notably, ZSH‐2208 exhibited IC_50_ values of 0.61 µM for KYSE150‐TRCs and 0.96 µM for EC109‐TRCs (Figure 2D,E). In contrast, the IC_50_ values for KYSE150 and EC109 cells increased to 2.20 and 2.40 µM, respectively, representing a 2.5 to 3.7 fold increase compared with IC_50_ values of TRCs for ZSH‐2208 (Figure S1B). Clone formation assays and clonesphere formation assays were conducted to further investigate the impact of ZSH‐2208 on tumour cell proliferation, the results indicated that ZSH‐2208 significantly reduced the number of ESCC cells and inhibited the growth of ESCC‐TRCs clonal spheres at a concentration of 1 µM, as evidenced by the size of the clonal spheres. This finding demonstrates that ZSH‐2208 exerts a marked inhibitory effect on the growth of both ESCC cells and ESCC‐TRCs at this low concentration (Figures 2F,G and S3A–D). Indicating that the ability of ZSH‐2208 to hinder ESCC‐TRCs growth by substantially reducing clone sphere diameter and volume. Flow cytometry were subsequently used to investigate the apoptosis‐inducing potential of ZSH‐2208. The results demonstrated that ZSH‐2208 significantly increases apoptosis rate in KYSE150‐TRCs from 4.08 to 8.38% and in EC109‐TRCs from 31.40 to 43.10% at a concentration of 1 µM, with a marked enhancement observed at a concentration of 5 µM (Figures 2H,I and S3E–H). And the immunofluorescence experiments revealed increased cleaved‐Caspase3 levels and decreased Ki67 levels following ZSH‐2208 treatment (Figure S3I–K). Overall, these findings support the potential of ZSH ‐2208 to inhibit proliferation and induce apoptosis in both ESCC cell lines and ESCC‐TRCs. Transwell migration and invasion assays were conducted to examine the impact of ZSH‐2208 on the migration and invasion of ESCC cells and ESCC‐TRCs. Results revealed a noticeable reduction in the number of successfully migrating and invading ESCC cells and ESCC‐TRCs in the ZSH‐2208 treatment group compared with the DMSO group (0.1% DMSO‐containing medium), indicating inhibition of migration and invasion ability (Figures 2J,K and S3L–Q). Furthermore, cell scratch assay conducted further validate ZSH‐2208's inhibitory effect on the migration ability of adhesive KYSE150 cells (Figure S3R,S). Additionally, the expression of CD44 in KYSE150‐TRCs and EC109‐TRCs was dose‐dependently suppressed by ZSH‐2208, indicating its potential to suppress stemness (Figure 2L). Flow cytometry was also employed to explore the influence of ZSH‐2208 on the cell cycle of ESCC cells and ESCC‐TRCs. The outcomes indicated that ZSH‐2208 solely disrupted the cell cycle of EC109‐TRCs, arresting them in the G1 phase. While not observed in KYSE150, EC109 and KYSE150‐TRCs (Figures 2M–O and S3T).

**FIGURE 3:**
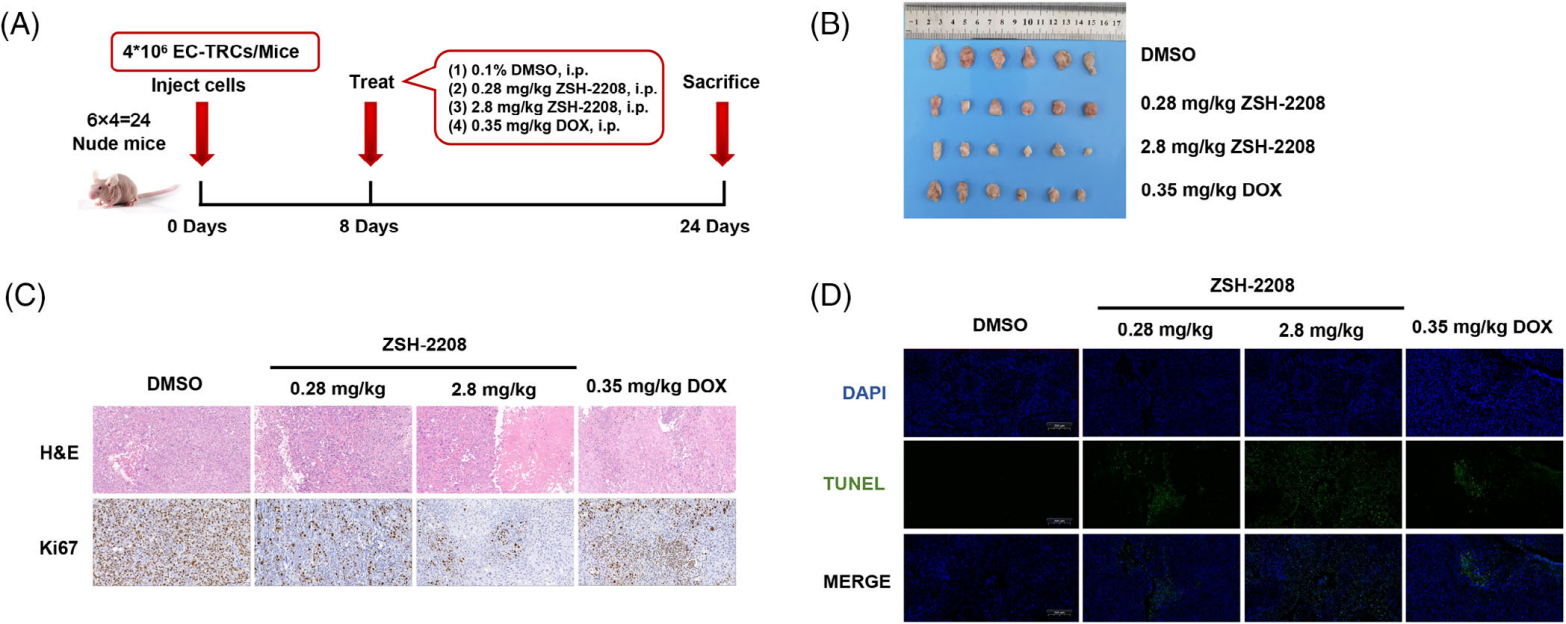
ZSH‐2208 inhibits the growth of ESCC‐TRCs in vivo. (A) Dosing regimen for subcutaneous tumour formation assay in nude mice. (B) Dissection for subcutaneous tumours following nude mouse sacrifice. (C) Haematoxylin and eosin (HE) staining of tumour tissue. (D) Immunohistochemistry staining for proliferation marker Ki67.

The results of in vivo assessment showed that a low dose (0.28 mg/kg) of ZSH‐2208 significantly inhibited subcutaneous tumour growth in nude mice, as evidenced by the reduction in tumour weight and volume (Figures 3A,B and S4A,B). With the high‐dose group (2.8 mg/kg ZSH‐2208) exhibiting significantly lower tumour weight compared with the positive control group (0.35 mg/kg DOX). The tumour tissue sections were stained with H&E. The results of immunohistochemical staining and TUNEL immunofluorescence staining demonstrated the molecular impact of ZSH‐2208 on ESCC‐TRCs growth in vivo, indicating that 0.28 mg/kg of ZSH‐2208 significantly reduced Ki67 expression and increased the proportion of fluorescence‐positive cells in the TUNEL assay (Figures 3C,D and S4C,D). Importantly, the in vivo experimental results confirmed that ZSH‐2208 inhibited ESCC‐TRCs proliferation and promoted apoptosis, which was consistent with the in vitro findings. Additionally, histological sectioning and H&E staining of key organs (heart, liver, spleen, lung and kidney) collected from each group showed no adverse effects of ZSH‐2208 during subcutaneous tumour formation (Figure S4E). Furthermore, there were no significant changes in the body weight of nude mice across all treatment groups compared with the 0.1% DMSO treatment group (Figure S4F), indicating the safety of ZSH‐2208 as a therapeutic agent.

In summary, our study thoroughly examined and evaluated the inhibitory effects of ZSH‐2208 on the growth of ESCC‐TRCs through both in vitro and in vivo experiments. The results demonstrate the potent efficacy of ZSH‐2208 in suppressing proliferation, migration and invasion, while promoting apoptosis. Furthermore, ZSH‐2208 significantly inhibited the growth of subcutaneous ESCC tumours in nude mice.

### 2.3 ZSH‐2208 inhibits the transcription of TNFAIP3 through reducing the expression of protein of RARγ

**FIGURE 4:**
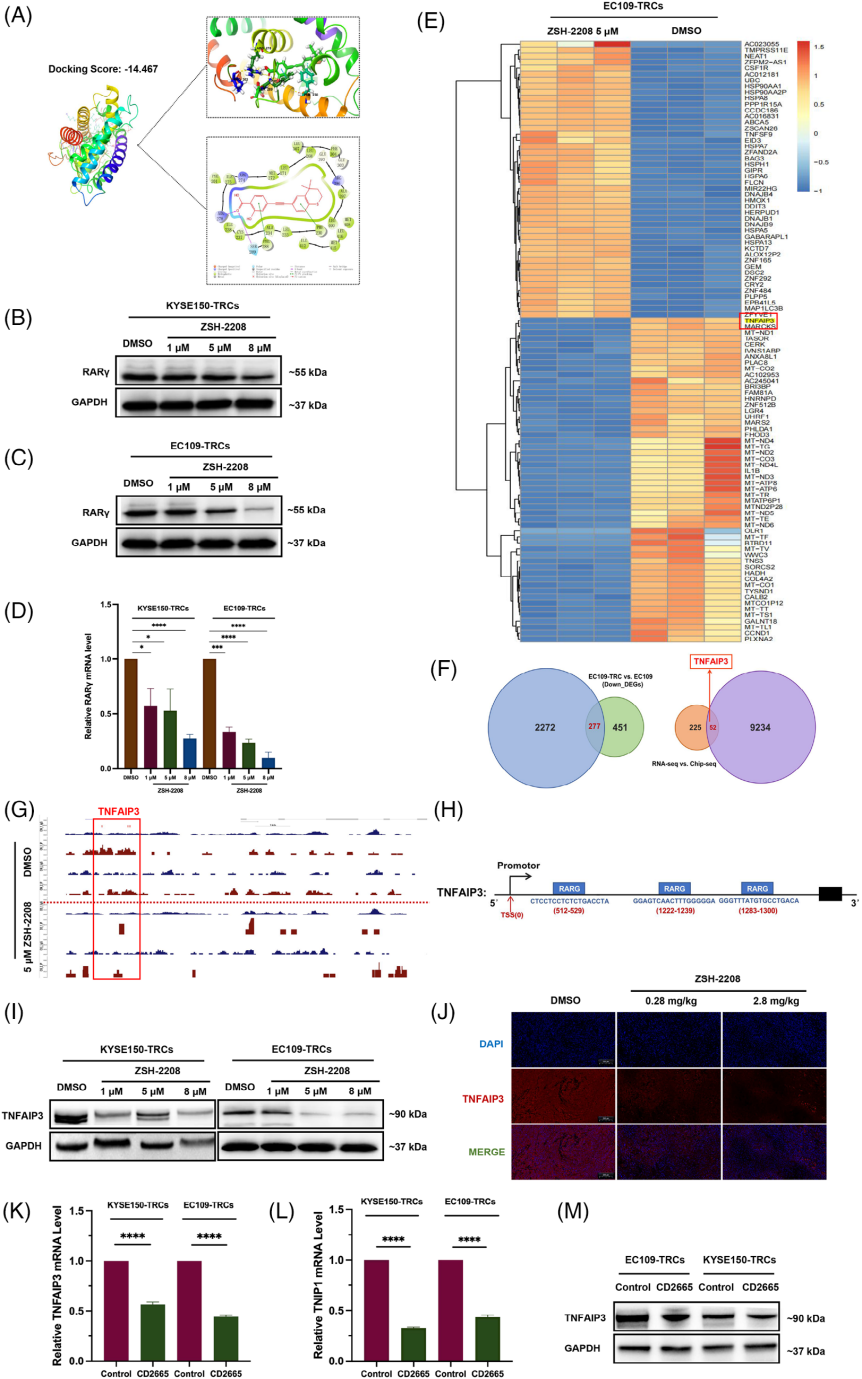
ZSH‐2208 inhibits the transcription of TNFAIP3 through reducing the expression of protein of RARγ. (A) Pattern diagram of molecular docking simulation of ZSH‐2208 to RARγ protein. (B–D) Effect of ZSH‐2208 on RARγ protein and gene expression levels in ESCC‐TRCs. (E) Heatmap of the significantly upper (lower)—modulated differentially expressed gene (DEGs) Top50 after ZSH‐2208 treatment. (F) Combined analysis of sequencing by RNA‐seq and ChIP‐seq identified TNFAIP3, a key effector molecule for ZSH‐2208. (G) ZSH‐2208 treatment significantly reduced the binding peak of RARγ protein in the promoter region of TNFAIP3 gene. (H) Three possible binding sites for the RARγ protein on the TNFAIP3 gene promoter. (I) Effect of ZSH‐2208 on TNFAIP3 and TNIP1 at protein level in ESCC‐TRCs. (J) Immunofluorescence staining of TNFAIP3 protein was performed in subcutaneous tumour tissues of nude mice to observe the effect of ZSH‐2208 on TNFAIP3 protein expression. DOX, doxorubicin; red, TNFAIP3: blue. (K–M) Effect of CD2665 on TNFAIP3 and TNIP1 at gene and protein level in ESCC‐TRCs (*p < .05, **p < .01, ***p < .001, ****p < .0001; ns, not significant).

Initially, we investigated the regulatory influence of ZSH‐2208 on RARs. The chemical structure of ZSH‐2208 resembles several known pan‐antagonists of RARs, such as ATRA and acyclic retinoid (ACR). Molecular docking simulations were used to explore the three‐dimensional binding interaction between ZSH‐2208 and the RARγ protein, revealing a strong binding affinity between ZSH‐2208 and RARγ (Figure 4A). The subsequent qRT‐PCR and Western blot analyses revealed that ZSH‐2208 significantly reduced RARγ expression in KYSE150‐TRCs and EC109‐TRCs, both at the protein and gene levels (Figure 4B,C,D). Notably, the results showed a dose‐dependent inhibition of RARγ by ZSH‐2208. However, the expression levels of RARα and RARβ remain unchanged with increasing concentrations of ZSH‐2208 (Figure S5A–D). Only RARγ expression in ESCC‐TRCs is suppressed by ZSH‐2208 in a dose‐dependent manner. Therefore, we can conclude that ZSH‐2208 acts as a modulator of RARγ in a dose‐dependent manner.

Further investigation was conducted to elucidate the function of RARγ, targeted by ZSH‐2208, as a transcription factor in ESCC. Transcriptome sequencing (RNA‐seq) was performed on EC109‐TRCs and EC109 to identify significantly differentially expressed genes (DEGs) within the DMSO and ZSH‐2208 treatment groups. The top 50 up‐regulated and down‐regulated DEGs following ZSH‐2208 treatment in EC109‐TRCs are presented with a heatmap (Figures 4E and S5E). The down‐regulated genes after anti‐tumour drug treatment, which are often involved in oncogenesis, were identified by intersecting the significant down‐regulated DEGs in both EC109‐TRCs and EC109. This resulted in a curated gene set consisting of 277 genes (Figure 4F). Meanwhile, ChIP‐seq sequencing was performed on EC109‐TRCs to directly detect changes in downstream genes that interact with RARγ protein upon treatment with DMSO or ZSH‐2208. The results revealed significant alterations in the expression of genes tightly bound to RARγ protein following ZSH‐2208 treatment, which correlated with decreased RARγ expression. Subsequently, this subset of genes was intersected with the previous 277 DEGs to obtain a final gene set consisting of 52 genes (Figure 4F). The analysis revealed that TNFAIP3, one of the significantly altered genes in DEGs, may directly binds to RARγ protein and is down‐regulated after ZSH‐2208 treatment. Previous studies have shown that high expression of TNFAIP3 is associated with a low survival rate in ESCC patients.^14^ Therefore, we speculate that TNFAIP3 may function as an oncogene in ESCC. Further analysis have shown that the gene sequence of TNFAIP3 aligns with the RARγ protein motif (Figures 4G and S5F), and ZSH‐2208 treatment significantly reduces RARγ protein binding in the TNFAIP3 promoter region. Additionally, the TNFAIP3 gene promoter proportion within peak‐annotated genomic functional elements notably decreased from 3.29 to 2.74% (Figure S5G), and three RARγ protein binding sites were identified in the TNFAIP3 promoter region (Figure 4H). We used primers designed based on the TNFAIP3 gene promoter sequence that directly binds to RARγ protein for QRT‐PCR, and the results showed a significant reduction of TNFAIP3 in the ZSH‐2208 treatment group (Figure S5H).

The expression of TNFAIP3 at both the protein and gene levels in ESCC‐TRCs treated with ZSH‐2208 showed a decreasing trend, as well as its binding protein TNIP1, with increasing doses of ZSH‐2208 (Figures 4I and S5I,J), which is consistent with the dose‐dependent inhibitory effect of ZSH‐2208 on RARγ observed in previous in vitro experiments. The TNFAIP3 protein was immunofluorescently stained in tumour tissue sections from animal experiments to observe it change after ZSH‐2208 treatment in subcutaneous tumours of nude mice. The results showed a significant decrease in TNFAIP3 levels (Figures 4J and S5K). These findings were consistent with the results obtained from in vitro experiments. Additionally, we treated ESCC‐TRCs with CD2665 (MCE, HY‐107437, USA), a selective antagonist of RARγ, and observed a reduction in the expression levels of both TNFAIP3 and TNIP1 at the gene level. This finding indicates that the decrease in TNFAIP3 expression following ZSH‐2208 treatment is indeed mediated through RARγ (Figure 4K,L,M). In summary, we concluded that ZSH‐2208 inhibits the transcriptional activation of TNFAIP3 regulated by the RARγ protein by down‐regulating its expression.

We also investigated the differential expression of TNFAIP3 in cancerous and adjacent non‐cancerous tissues of EC patients using the TCGA database analysis tool, which was developed by Peking University and is available at http://gepia.cancer‐pku.cn. The results indicated a significant difference in TNFAIP3 expression between these tissue types, which the expression of TNFAIP3 is significantly higher in cancerous tissues compared with adjacent non‐cancerous tissues in EC patients (*p* < .05) (Figure S5L). Furthermore, the correlation between patient survival and TNFAIP3 protein expression in tumour tissues was evaluated using survival analysis in R version 4.3.0 software with data from the TCGA database (Supporting Information). The preprocessed RNA‐seq data and corresponding clinical information of EC were downloaded from TCGA data portal. The clinical samples from Esophagus (squamous cell neoplasms and adenomas and adenocarcinoma) were selected, while samples from other anatomic sites such as cystic, mucinous and serous neoplasms were excluded. A total of 172 EC patients with detailed follow‐up time were included for subsequent analysis. Results showed that high TNFAIP3 expression is positively correlated with poor survival in ESCC patients (*p* < .05) (Figure S5M). Therefore, it can be inferred that TNFAIP3 is a key effector of ZSH‐2208, which inhibits the growth of ESCC‐TRCs by indirectly reducing its expression through the inhibition of RARγ protein expression.

### 2.4 The clinic significance of TNFAIP3 in ESCC

**FIGURE 5:**
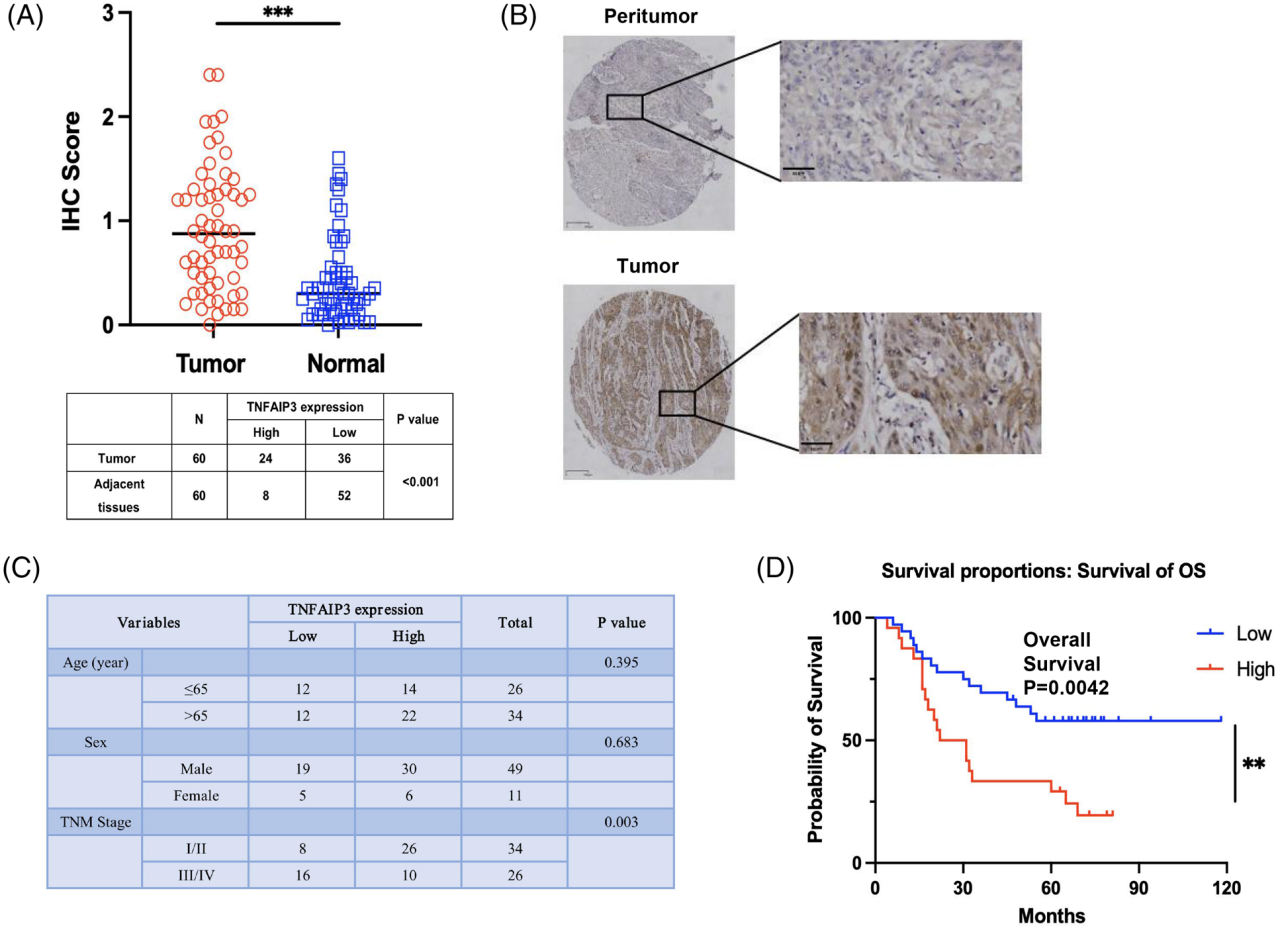
The clinic validation of TNFAIP3. (A and B) Differential expression of TNFAIP3 protein in cancer and adjacent non‐cancerous tissues of ESCC patients. (C) Chi‐square test was used to correlate TNFAIP3 expression levels with clinically relevant parameters in ESCC patients. (D) Kaplan–Meier survival analysis and Log‐Rank statistical test were used to correlate TNFAIP3 expression levels with overall survival (OS) in ESCC patients (**p < .01, ***p < .001).

We investigated the clinical significance of TNFAIP3 in a cohort of ESCC patients, collecting tissue samples from tumours and adjacent regions from 60 patients, who had comprehensive clinicopathological and follow‐up data from Zhongshan Hospital, Fudan University. These samples, which were integrated into a tissue microarray (TMA), were evaluated for TNFAIP3 protein levels using immunohistochemical staining. We found that the expression of TNFAIP3 was significantly higher in tumour tissue compared with adjacent tissue (Figure 5A,B,C). The Chi‐square test results showed no significant variation in TNFAIP3 expression across different age groups or genders. However, there was a significant association between TNFAIP3 expression and TNM stage. Kaplan–Meier survival analysis and Log‐Rank tests were conducted to correlate TNFAIP3 expression levels with overall survival (OS) in ESCC patients. Interestingly, the results revealed a significant correlation between low TNFAIP3 protein expression and improved prognosis in ESCC (Figure 5D). COX regression model was used to evaluate the impact of various factors on the survival of ESCC patients. The results of the univariate analysis indicated that both the expression level of TNFAIP3 and the TNM stage independently predicted prognosis in ESCC patients (Table 1), which further supports the potential of targeting TNFAIP3 expression as a promising strategy for treating these patients.

**TABLE 1:** COX univariate and multivariate analysis of prognostic factors related to overall survival (OS) in patients with ESCC (*n* = 60).

Variables	Univariate analysis				Multivariate analysis			
	*p* value	HR	95% CI		*p* value	HR	95% CI	
			Lower limit	Upper limit			Lower limit	Upper limit
High level of TNFAIP3 in tumour	**.022** *	2.709	1.155	6.353	**.029** *	2.742	1.111	6.766
Gender	.889	0.939	0.388	2.272				
Age	.387	1.022	0.973	1.074				
CEA	.558	1.059	0.875	1.281				
CA199	.686	1.003	0.989	1.017				
TNM stage	**<.001** *	8.757	4.599	16.677	**<.001** *	10.023	4.991	20.130

^*^Statistically significant (*p* < .05).

COX regression risk model was used to analyse the effects of each factor on the overall survival of patients (*p < .05).

### 2.5 TNFAIP3 was a key effector molecule of ZSH‐2208 in ESCC treatment

**FIGURE 6:**
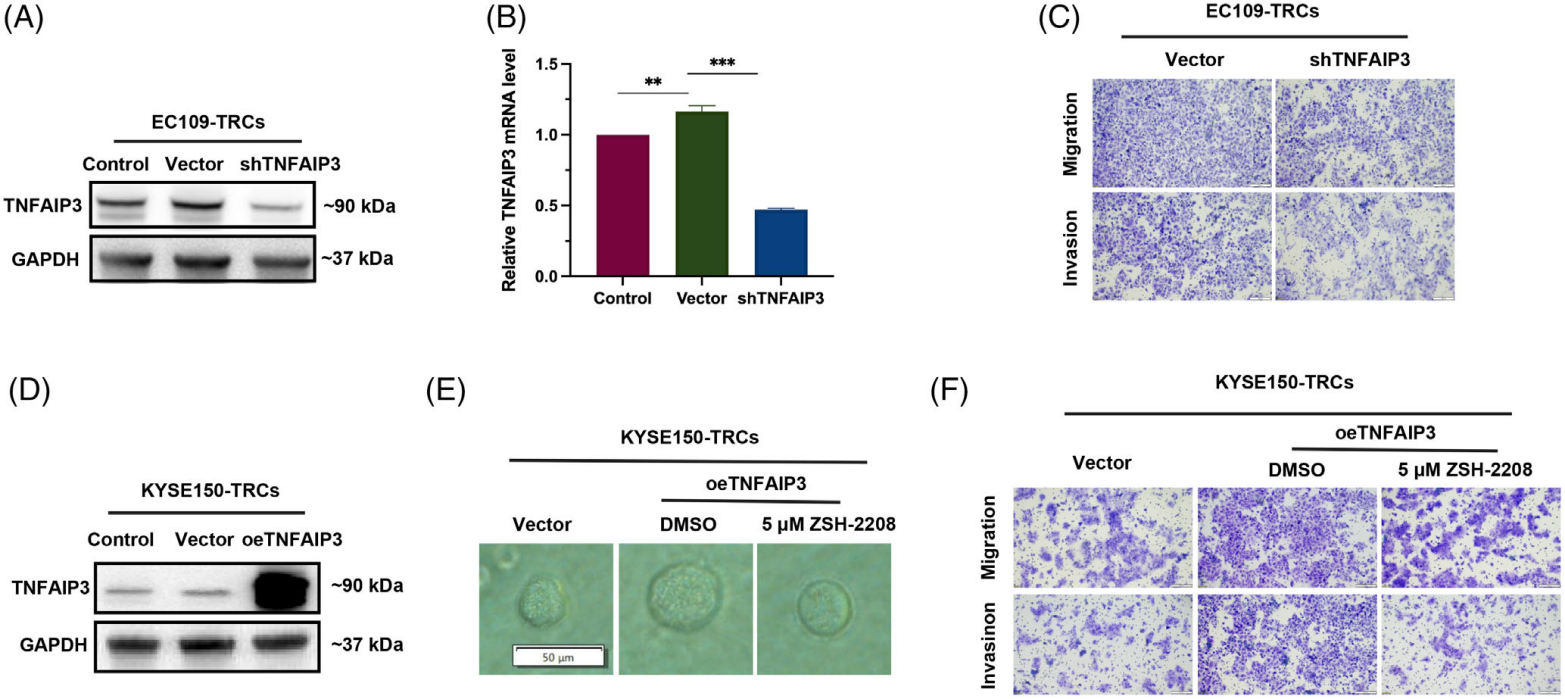
TNFAIP3 serves as a crucial effector molecule of ZSH‐2208 in ESCC‐TRCs. (A and B) TNFAIP3 expression was significantly reduced in shTNFAIP3 EC109‐TRCs. Control: common cells; vector: vector control; shTNFAIP3: TNFAIP3 knockdown. (C) Effect of knockdown of TNFAIP3 on migration and invasion of EC109‐TRCs. (D) TNFAIP3 expression was significantly overexpressed in oeTNFAIP3 EC109‐TRCs. Control: common cells; vector: vector control; oeTNFAIP3: TNFAIP3 overexpression. (E and F) The promoting effect of TNFAIP3 overexpression on clonal sphere formation in KYSE150‐TRCs was reversed by ZSH‐2208 (**p < 0.01; ***p < 0.001).

To validate our hypothesis, we performed knockdown and overexpression of TNFAIP3 in ESCC cells and assessed the resulting changes in the biological functions of ESCC‐TRCs. Lentiviruses with TNFAIP3 knockdown were produced using siRNAs for the knockdown experiments. EC109 cells were then infected with these lentiviruses to establish shTNFAIP3 EC109. Results showed that siRNA1 had a higher knockdown efficiency than siRNA2 (Figure S6A,B,C,D). Additionally, TNFAIP3 knockdown EC109‐TRCs were cultured using a 3D soft fibrin gel derived from shTNFAIP3 EC109 (Figure 6A,B). The results of Transwell assays demonstrated that TNFAIP3 knockdown significantly impaired the migration and invasion of EC109‐TRCs and EC109 cells (Figures 6C and S6E,F). Flow cytometry analysis revealed that TNFAIP3 knockdown markedly increased the proportion of apoptotic cells in both EC109‐TRCs and EC109 cells (Figure S6G,H,I). Notably, a significant number of cells in the early stages of apoptosis were observed in both vector and TNFAIP3 knockdown group, likely due to the effect of lentiviral infection on EC109 growth.

Transgenic oeTNFAIP3 KYSE150 were generated by lentiviral infection to overexpress TNFAIP3, and subsequently cultured as oeTNFAIP3 KYSE150‐TRCs (Figures 6D and S6J). Clonesphere formation assays revealed that TNFAIP3 overexpression significantly enhanced the diameter and volume of KYSE150‐TRCs, indicating a promotion in their proliferation. Notably, the administration of 5 µM ZSH‐2208 partially reversed the proliferation‐promoting effect of TNFAIP3 overexpression on clonal spheres of KYSE150‐TRCs, indicating the ability of ZSH‐2208 to counteract this effect (Figures 6E and S6K). The overexpression of TNFAIP3 significantly increased the migration and invasion capacities of KYSE150‐TRCs, as demonstrated by Transwell assays. However, treatment with 5 µM ZSH‐2208 effectively counteracted this promoting effect (Figures 6F and S6L,M).

Overall, the knockdown of TNFAIP3 significantly affects EC109‐TRCs by promoting cell apoptosis and inhibiting migration and invasion, effects that are similar to those induced by ZSH‐2208 treatment. Additionally, the overexpression of TNFAIP3 enhances the proliferation, migration and invasion of KYSE150‐TRCs, confirming our earlier speculation that TNFAIP3 is the key molecule responsible for the growth‐inhibitory effect of ZSH‐2208 on ESCC‐TRCs.

## 3 DISCUSSION

RA, which is essential for embryonic development and human physiology during cellular growth and differentiation, plays a crucial role in regulating pluripotent effects. Extensive research has shown that the disruption of the RA signaling pathway is crucial in the development of various haematological and non‐haematological malignancies, including leukaemia, lung cancer and glioblastoma, among others,^15^ all of which are intricately linked to tumourigenesis. Consequently, targeting the RA signaling pathway represents an innovative approach for cancer prevention and treatment. In reality, RA, particularly ATRA, has shown significant therapeutic efficacy in acute promyelocytic leukaemia (APL).^16^ ATRA is incorporated into various anti‐cancer regimens, including those for squamous cell carcinoma of the head and neck, ovarian cancer, bladder cancer and neuroblastoma.^17^ ATRA also has significant therapeutic potential in digestive tract tumours, including GC. For example, ATRA‐adjuvanted mRNA vaccines can induce anti‐tumour immune responses in CRC mucosa. And the mechanism involves co‐delivering ATRA and mRNA through lipid nanoparticles (LNP) to stimulate intestinal mucosal immune responses.^18^ Additionally, retinoids and immune checkpoint inhibitors (ICIs) could be potential combinations for treating GC by selecting patients sensitive to ATRA. ESCC is one of malignant gastrointestinal cancers. In the clinical treatment of ESCC, ATRA and its analogues have not been utilised as primary therapeutic agents, and there is a limited number of studies exploring the potential advantages of ATRA for patients with ESCC. Li et al.^19^ demonstrated that ATRA inhibits tumour growth in ESCC and enhances the responsiveness of ESCC tumours to anti‐PD‐1 immunotherapy by promoting the translation of OTUD6B. Therefore, the combination of ATRA and anti‐PD‐1 ICIs may provide therapeutic benefits for patients with ESCC.^19^ Lu et al.^20^ demonstrated that ATRA significantly inhibited the growth of EC9706 xenografts in nude mice, exhibiting notable anti‐tumour effects. The underlying mechanism may involve the specific down‐regulation of VEGF signaling pathway by ATRA, thereby inhibiting neovascularisation in ESCC cells.^20^ Currently, ATRA is primarily utilised in the clinical treatment of ESCC patients as an adjunct to chemotherapy. However, its limited water solubility poses challenges for formulating it for parenteral administration, thereby impeding its practical application in clinical settings.^17^ Furthermore, the potential teratogenic effects of RA significantly restrict its use as a therapeutic option for women of reproductive age.^21^ Therefore, it is crucial to overcome these limitations and expand the patient population that can benefit from effective treatment by developing novel retinoids.^22^

In previous studies, our team introduced WYC‐209, an innovative RA analogue that effectively inhibits the growth and metastasis of TRCs in malignancies such as melanoma and liver cancer, both in vitro and in vivo, without causing significant toxic side effects. To enhance the therapeutic potential of RA analogues and expand the repertoire of agents available for anti‐tumour therapy, our team meticulously designed and developed a series of novel RA analogues derived from WYC‐209. We systematically screened these newly synthesised RA analogues and ultimately identified ZSH‐2208, which exhibited potent growth inhibition in ESCC cells and ESCC‐TRCs.

Results also showed that ZSH‐2208 significantly inhibited the growth of ESCC cell lines and ESCC‐TRCs better than ATRA or doxorubicin, and it displayed a lower IC_50_ value in ESCC‐TRCs compared with ESCC cells. In addition, ZSH‐2208 exhibits superior water solubility and reduced toxic side effects compared with ATRA. These findings underscore the promising anti‐cancer potential of ZSH‐2208, a novel RA analogue, in the treatment of ESCC. Study has showed that the role of CSCs in the progression, drug resistance and recurrence of ESCC is critical.^4^ In our study, ESCC‐TRCs demonstrated reduced sensitivity to DOX, suggesting a potential development of drug resistance following treatment.^14^ However, ZSH‐2208 significantly reduced the gene expression levels of CD44, a recognised stemness marker in ESCC‐TRCs, which suggest that ZSH‐2208 may exhibit enhanced efficacy against CSCs. Given the significant toxicity and resistance associated with conventional chemotherapeutic agents, such as DOX,^1^ novel RA analogues present the potential to effectively inhibit tumour cell growth, thereby surpassing the limitations of traditional chemotherapy. In summary, innovative RA analogues, including WYC‐209 and ZSH‐2208, have demonstrated their ability not only to inhibit the proliferation of various tumour cell types but also to exert a more potent inhibitory effect on TRCs that possess stemness characteristics. This emerging class of RA analogues presents a targeted and novel strategy to overcome tumour resistance driven by CSCs, representing a promising advancement in the ongoing fight against malignancies.

The primary treatment for ESCC is surgery, while the incorporation of chemoradiotherapy or perioperative chemotherapy has significantly improved patient survival rates.^14^ Nevertheless, it remains essential to identify effective adjuvant therapies for patients diagnosed with ESCC. Our investigation, which focused on the growth‐inhibitory effects of ZSH‐2208 on cancer cells, yielded compelling results. Notably, ZSH‐2208 effectively reducing proliferation, migration and invasion while promoting apoptosis in ESCC‐TRCs in vitro. Encouragingly, our in vivo experiments demonstrated that ZSH‐2208 effectively restrained the growth of subcutaneous tumours induced by ESCC‐TRCs in nude mice. In summary, ZSH‐2208 exhibits therapeutic promise for patients suffering from ESCC. ZSH‐2208 specifically targets RARγ, a subtype of the RAR family. Our study included molecular docking simulations that conclusively demonstrate strong binding affinity of ZSH‐2208 to the RARγ protein. The oncogenic potential of RARγ, which may arise from its selective expression, can directly modulate key target genes and disrupt their expression, leading to a ripple effect on various cellular behaviours.^23^ Prior studies have highlighted the overexpression of RARγ in human CRC tissues and multiple CRC cell lines, findings that suggest an increase in sensitivity to chemotherapeutic agents and a potential counteraction of multidrug resistance mechanisms.^24^ Additionally, RARγ overexpression is associated with poor prognosis and chemoresistance in CCA patients.^25^ Furthermore, RARγ overexpression has been observed in primary tissue samples and cell lines derived from HCC and approximately half of CCRCC patients,^26^, ^27^ indicating its potential as a target for anti‐tumour therapy. However, the relationship between RARγ expression levels in tumours and ESCC patient survival and prognosis remains unexplored territory that requires further investigation. In summary, the involvement of RARγ in malignancies and its potential as a therapeutic target have been confirmed across various contexts, emphasising its significance for anti‐tumour interventions. Importantly, our research has demonstrated the promising therapeutic potential of ZSH‐2208 in ESCC treatment.

In our investigation, initial explorations revealed that ZSH‐2208 modulates specific subtypes of the RARs family and primarily functions as a dose‐dependent modulator of RARγ. Further analysis demonstrated that the expression of TNFAIP3 is down‐regulated following treatment with ZSH‐2208. Importantly, TNFAIP3 was found to play a crucial role in this network by significantly interacting with the RARγ protein, which directly binds to the promoter region of TNFAIP3 and participates in its transcriptional regulation. Additionally, our study found that CD2665, one of the RARγ antagonist, inhibited TNFAIP3 expression in ESCC‐TRCs, indicating that TNFAIP3 may function as a downstream target regulated by RARγ. TNFAIP3, a key player in the human immune system, exhibits a dual role in cancer biology. Initially identified as a tumour suppressor gene in CRC, HCC and other cancers, TNFAIP3 was later shown to promote tumour cell growth and dissemination in BC, GC, melanoma and various other malignancies. Its involvement in carcinogenesis is associated with its ability to enhance the proliferation, migration and invasion of tumour cells while inhibiting apoptosis.^28^ In BC, the anti‐apoptotic mechanism of TNFAIP3, which involves its interaction with the protein A20 and HSP70, serves to protect BC cells from TNF‐induced cell death. Moreover, TNFAIP3 is pivotal in BC metastasis and significantly contributes to the TGF‐β1‐induced epithelial–mesenchymal transition (EMT).^29^ In GC cells, the elevated expression of TNFAIP3 protein is inversely correlated with the methylation of specific CpG sites located within its intronic region.^30^ The suppression of TNFAIP3 expression inhibits EMT, subsequently reducing the proliferation, migration and invasion of GC cells.^31^ In melanoma, the orchestrated deletion of TNFAIP3 significantly diminishes melanoma cell proliferation and tumour growth in mouse models. Conversely, the overexpression of TNFAIP3 promotes EMT in vitro and facilitates tumour metastasis in vivo.^32^

Our study showed that ZSH‐2208 reduced the expression of TNFAIP3 and effectively inhibited the growth of ESCC‐TRCs by suppressing proliferation, migration and invasion while promoting apoptosis. Our research identifies TNFAIP3 as a potential oncogene in ESCC, emphasising its potential as a therapeutic target. Analysis of EC patient data from the TCGA database revealed significant differences in TNFAIP3 expression between malignant and non‐malignant tissues, and higher expression levels associated with poorer survival outcomes for patients. These findings reinforce the association between TNFAIP3 expression and clinical outcomes in ESCC patients, suggesting its relevance as a therapeutic target. In this context, our investigation convincingly hypothesises that TNFAIP3 serves as a key mediator in the growth‐inhibitory effect of ZSH‐2208 on ESCC‐TRCs. ZSH‐2208 reduces RARγ protein levels, which in turn suppresses the transcriptional expression of TNFAIP3. This assertion is corroborated by experimental validations indicating that the knockdown or overexpression of TNFAIP3 in ESCC‐TRCs yields contrasting outcomes: knockdown diminishes migration and invasion while enhancing apoptosis, whereas overexpression promotes migration and invasion. Importantly, the administration of ZSH‐2208 reversed the proliferative effects associated with TNFAIP3 overexpression, further confirming the intricate relationship between ZSH‐2208 and TNFAIP3. Previous studies have demonstrated that sulforaphane inhibits the progression of ESCC by down‐regulating the expression of TNFAIP3 and PLAU in a p65‐dependent manner.^33^ In the case of ESCC‐TRCs, ZSH‐2208 may exert growth‐inhibitory effects by suppressing TNFAIP3 expression at the transcriptional level. Therefore, modulating TNFAIP3 expression levels presents a promising approach for the therapy of diverse cancers.

To further confirm the significance of regulating TNFAIP3 expression in the clinical treatment of patients with ESCC, our study employed TMAs derived from ESCC patients to evaluate TNFAIP3 protein levels in both cancerous and adjacent non‐cancerous tissues. Subsequently, we analysed the association between TNFAIP3 expression levels and patient prognosis. The results demonstrated a positive correlation between elevated TNFAIP3 protein expression and poor prognosis in ESCC patients, indicating that high TNFAIP3 expression serves as an independent factor linked to unfavourable survival outcomes. Therefore, we propose that TNFAIP3, a critical modulator of ZSH‐2208, may serve as a significant prognostic indicator for patients with ESCC and could also represent a potential target for anti‐tumour therapies. Previous studies have not established a connection between TNFAIP3 and RA (or RARs). Our investigation has revealed a direct interaction between the RARγ protein and the promoter region of the TNFAIP3 gene in ESCC‐TRCs, which serves as a determinant of transcriptional regulation. In this study, ZSH‐2208 inhibited the growth of ESCC‐TRCs by targeting the RARγ–TNFAIP3 axis. The combination of anti‐tumour strategies targeting RARs/RXRs with TNFAIP3 presents a novel avenue for managing ESCC, wherein ZSH‐2208 plays a pivotal role due to its inherent anti‐tumour activity and its potential to facilitate the development of innovative RA analogues in the anti‐tumour domain.

In conclusion, our study has identified ZSH‐2208 as a promising candidate for therapeutic intervention in ESCC. This RARγ modulator exhibits potent inhibitory effects on the growth of ESCC‐TRCs both in vitro and in vivo. Furthermore, our investigation has revealed the potential oncogenic role of TNFAIP3 in patients with ESCC. Notably, we have discovered a significant association between TNFAIP3 and the key transcription factor RARγ, a finding that introduces a novel avenue for developing targeted therapeutic strategies in the clinical management of ESCC.

Despite the progress made in our study, several shortcomings remain that warrant further investigation. In the context of animal experiments, we plan to conduct additional explorations, focusing on optimising both the dosage and administration methods to enhance the tumour‐inhibitory efficacy of ZSH‐2208 at lower doses. Furthermore, we will incorporate the patient‐derived tumour xenograft model, which closely mimics the conditions of actual patients, to validate the drug's efficacy and identify the optimal therapeutic dosage. At the same time, we require more concrete and intuitive evidence beyond simulated molecular docking to confirm the binding of ZSH‐2208 to RARγ and its direct regulation of RARγ, as simulated molecular docking is merely an in silico prediction and does not constitute real experimental evidence. We aim to demonstrate that ZSH‐2208 acts as a direct inhibitor or agonist of RARγ. Additionally, our current understanding of the mechanism of ZSH‐2208 is limited, necessitating further investigation into its detailed molecular mechanisms in ESCC‐TRCs and its apoptosis‐inducing effects on other types of EC stem cells like adenocarcinoma of esophagus. We hope to expand the potential therapeutic applications of ZSH‐2208 across different cancer types.

In future studies, we plan to use more realistic in vivo experimental models to validate the drug's efficacy and provide more detailed molecular biological evidence to elucidate the complete and specific molecular mechanisms through which ZSH‐2208 exerts its anti‐tumour effects by regulating RARγ. Ultimately, we aim to expand and validate the potential clinical applications of ZSH‐2208, bringing new therapeutic hope to patients with ESCC and other types of cancer.

In conclusion, our research underscores the potential of ZSH‐2208, a selective RARγ modulator, as a therapeutic agent for ESCC, particularly in targeting ESCC‐ TRCs. And ZSH‐2208 inhibits the growth of ESCC‐TRCs by targeting the RARγ–TNFAIP3 axis. Moreover, our research elucidates the oncogenic implications of TNFAIP3 in ESCC and its intricate relationship with the crucial transcription factor RARγ, thereby suggesting a promising avenue for future clinical interventions in the treatment of ESCC.

## 4 MATERIALS AND METHODS

### 4.1 Culture of ESCC‐TRCs

The establishment of ESCC‐TRCs involved sequential steps. Initially, fibrinogen (Salmon fibrinogen; Pfenex, SEA‐133, USA) and thrombin (Salmon thrombin; Pfenex, SEA‐135, USA) solutions were prepared by thawing them on ice and diluting Fibrinogen to 2 mg/mL and thrombin to 0.1 U in pre‐cooled T7 buffer. Following this, ESCC cell lines KYSE150, EC109 and TE1 were trypsinised, centrifuged and resuspended in RPMI 1640 medium. The cell concentration was adjusted, and a mixture was created by combining 500 µL of cell suspension with an equivalent amount of 2 mg/mL fibrinogen solution, aiming for a final fibrinogen concentration of 1 mg/mL. This mixture was placed in a 96‐well plate, with 1 µL of 0.1 U thrombin solution and 50 µL of the cell‐fibrinogen mixture added to each well's centre. After gentle mixing and careful avoidance of bubbles, the plate was incubated at 37°C for 30 min. Subsequently, complete medium was introduced onto the gel surface, and further incubation occurred in a 5% CO_2_ incubator at 37°C. Following 24–48 h of incubation, the gel was dissolved either by adding the intended drug or 0.8 IU of DispaseII (Yeasen Biotechnology; 40104ES80, China). Complete dissolution led to TRCs collection through centrifugation. This process enabled the generation of ESCC‐TRCs for subsequent experimental investigations.

### 4.2 Establishment of ESCC subcutaneous tumour models in nude mice

In optimal conditions and free of mycoplasma contamination, ESCC‐TRCs (4 × 10^6^) were injected into the upper left limb of 6‐week‐old male Balb/c mice for the in vivo metastasis assay. Regular observations were made on the mice's survival and subcutaneous tumour development. After 8 days, drug treatment commenced via the tail vein, utilising doses of 0.1% DMSO, 0.28 mg/kg ZSH‐2208, 2.8 mg/kg ZSH‐2208 and 0.35 mg/kg DOX hydrochloride. Tumour harvesting was conducted 24 days post‐injection, enabling the establishment of the ESCC subcutaneous tumour models.

### 4.3 Schrödinger molecular docking simulation

The three‐dimensional structure of RARγ, characterised by high resolution and ligand interaction, was obtained from the UniProt database (https://www.uniprot.org/) and downloaded from the RCSB Protein Data Bank (http://www.rcsb.org). Molecular docking of ZSH‐2208 with RARγ was conducted using Schrödinger software. The ZSH‐2208 structure was imported into Maestro, where water molecules were selectively removed and energy minimisation was performed using the Protein Preparation Wizard. Polymeric complexes were simplified, and ligand properties were optimised to ensure the correct chiral 3D structure. The Ligand Docking module facilitated molecular docking by defining the docking box and adjusting parameters. Ligands were loaded, and the results were analysed to determine binding interactions and affinities. Finally, PyMOL software was employed to visualise the docking structures in a cartoon format.

### 4.4 ESCC patients recruitment and follow‐up

Patient recruitment and tissue collection were conducted at Fudan University Affiliated Zhongshan Hospital after obtaining Institutional Review Board approval (Approval Letter No. B2021‐129). A total of 60 patients, aged 51–72 years, diagnosed with ESCC, were prospectively selected based on predefined inclusion and exclusion criteria. Patients were enrolled from the thoracic surgery department. Surgical resections were performed as part of routine treatment, and both cancerous and adjacent non‐cancerous tissue specimens were collected and immediately preserved in formalin. Following tissue processing, the samples were embedded in paraffin blocks. TMAs were constructed using a semi‐automated TMA oesophageal squamous carcinoma‐2, incorporating representative cores from both cancerous and non‐cancerous tissues. Comprehensive clinical and pathological data, encompassing patient demographics, histological subtype and pathologic stage, were meticulously extracted from patients' medical records and stored in a secure electronic database. Longitudinal follow‐up data, which included details on treatment modalities and outcomes, were meticulously recorded and maintained for subsequent analysis. To evaluate the influence of various clinical variables on patient outcomes, statistical analysis employed a COX regression risk model.

### 4.5 Statistical analysis

The experimental data were analysed using GraphPad Prism 8.0. Statistical analysis was performed with SPSS 22.0 (IBM, Armonk, NY, USA). Continuous variables are presented as mean ± SD. Paired *t*‐tests were used to compare normally distributed measurement data between two groups. For normally distributed data involving multiple groups, variance testing was conducted. The Wilcoxon rank sum test was applied for non‐normally distributed data. For more details, see Supporting Information.

## AUTHOR CONTRIBUTIONS

Xin Cao, Jiayun Hou and Quanlin Li conceived the project. Ruoxue Chen, Junjie Ni, Xuan Huang and Wenrui Zhao performed the research experiments and organised and prepared the manuscript. Ruoxue Chen, Jiayun Hou, Quanlin Li and Xin Cao revised the manuscript. All authors approved the submission of this manuscript.

## CONFLICT OF INTEREST STATEMENT

The authors declare that there is no conflict of interests.

## ETHICS STATEMENT

All animal protocols were approved by the Ethical Committee on Animal Experiments of Animal Care Committee of Zhongshan Hospital Fudan University Shanghai Cancer Center.

## Supporting information



Supporting information

Supporting information

## Data Availability

The data that support the findings of this study are available on request from the corresponding author upon reasonable request.
