# Supplementary material for: ZSH‐2208: A novel retinoid with potent anti‐tumour effects on ESCC stem cells via RARγ–TNFAIP3 axis
Source: Clin Transl Med. 2024 Dec 26;15(1):e70148. doi: 10.1002/ctm2.70148 (PMC11670471; doi:10.1002/ctm2.70148)
Supplement: Supplementary file 1 — Supporting information [file CTM2-15-e70148-s002.docx]

**Supplementary Figure**

**
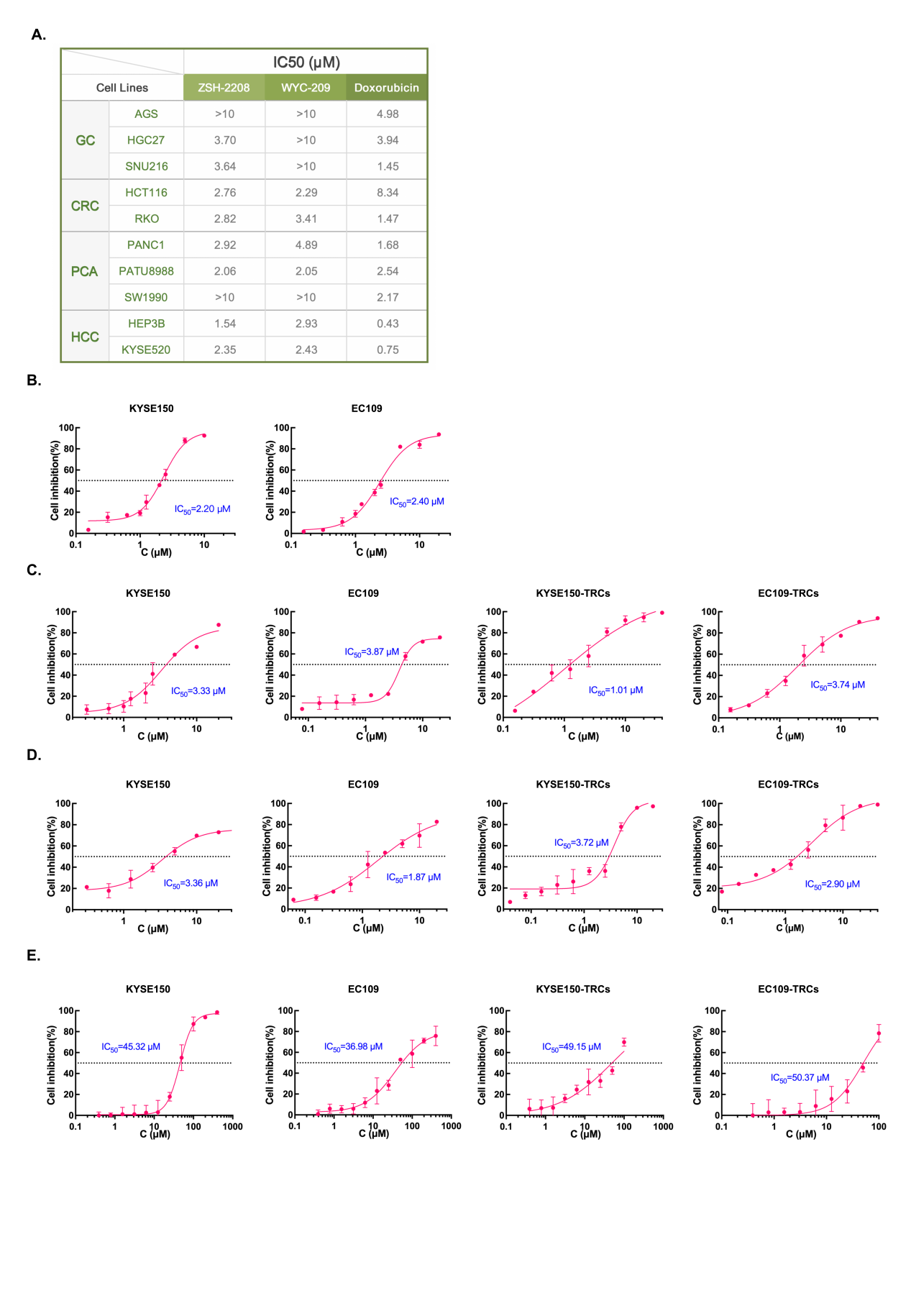
**

**Supplementary Figure 1.** A, IC_50_ values of ZSH-2208 on several type of tumor cell lines. B, IC_50_ of ZSH-2208 on KYSE150, KYSE150-TRCs, EC109 and EC109-TRCs. C, IC_50_ of WYC-209 on KYSE150, KYSE150-TRCs, EC109 and EC109-TRCs. D, IC_50_ of doxorubicin (DOX) on KYSE150 and EC109. E, IC_50_ of ATRA on KYSE150 and EC109.

**
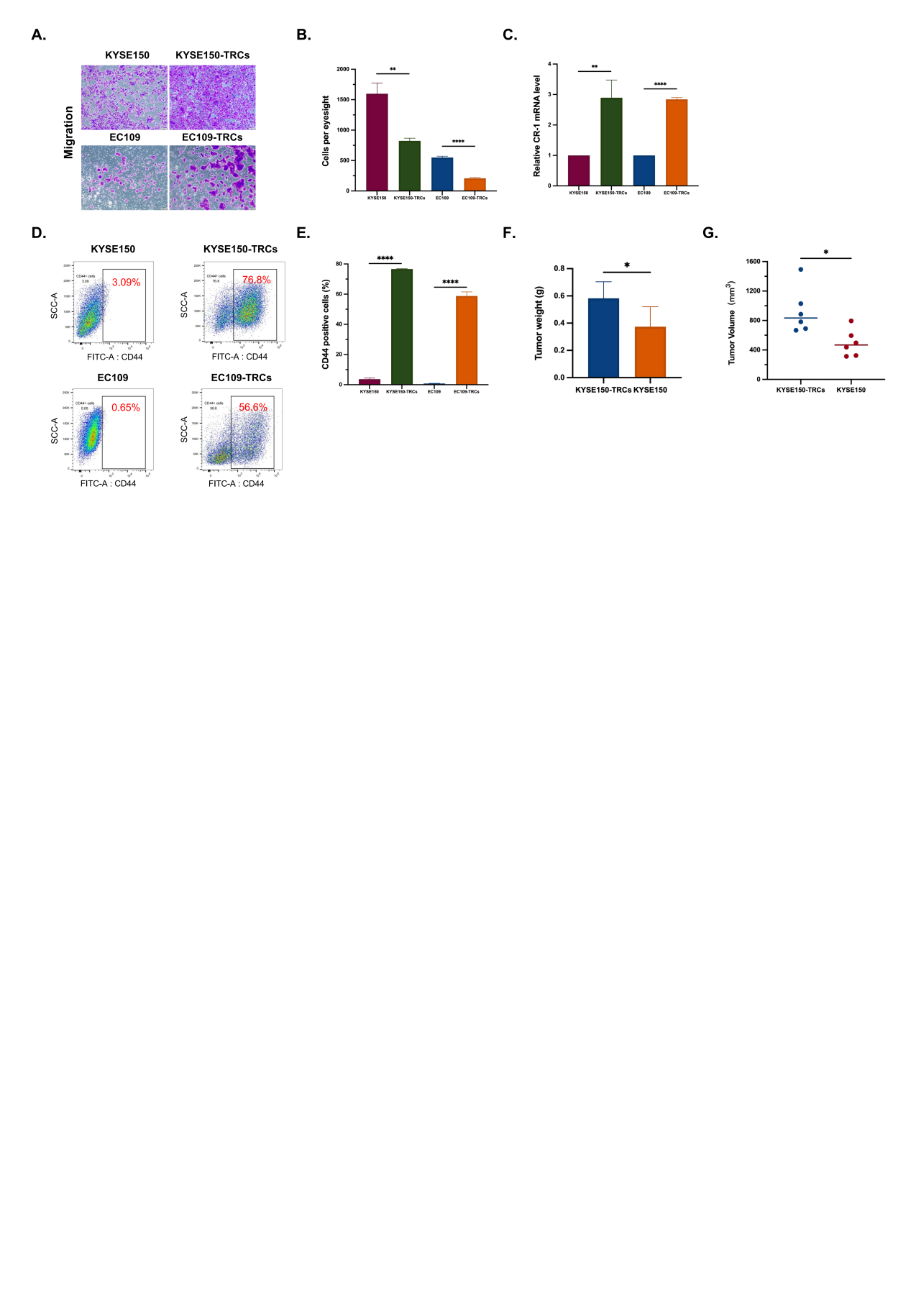
**

**Supplementary Figure 2.** A,B, Comparison of migration ability between ESCC cells and ESCC-TRCs. C. Expression of and CR-1 in ESCC-TRCs detected via qRT-PCR. D,E, Flow cytometry detects the proportion of CD44 positive cells in ESCC-TRCs. F,G, Weight and volume of subcutaneous tumor in nude mice. (*P < 0.05, ****P < 0.0001)

**
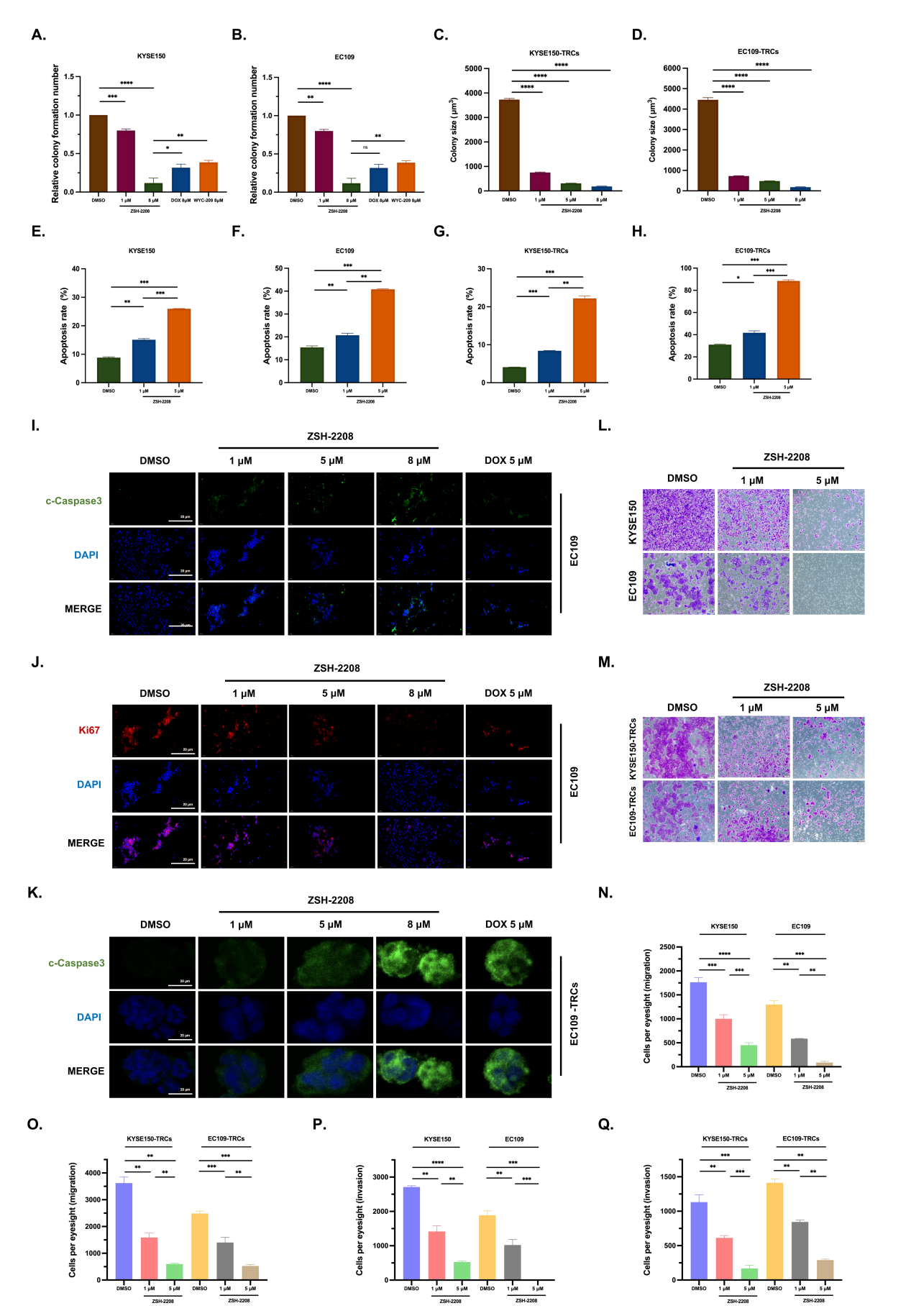
**

**
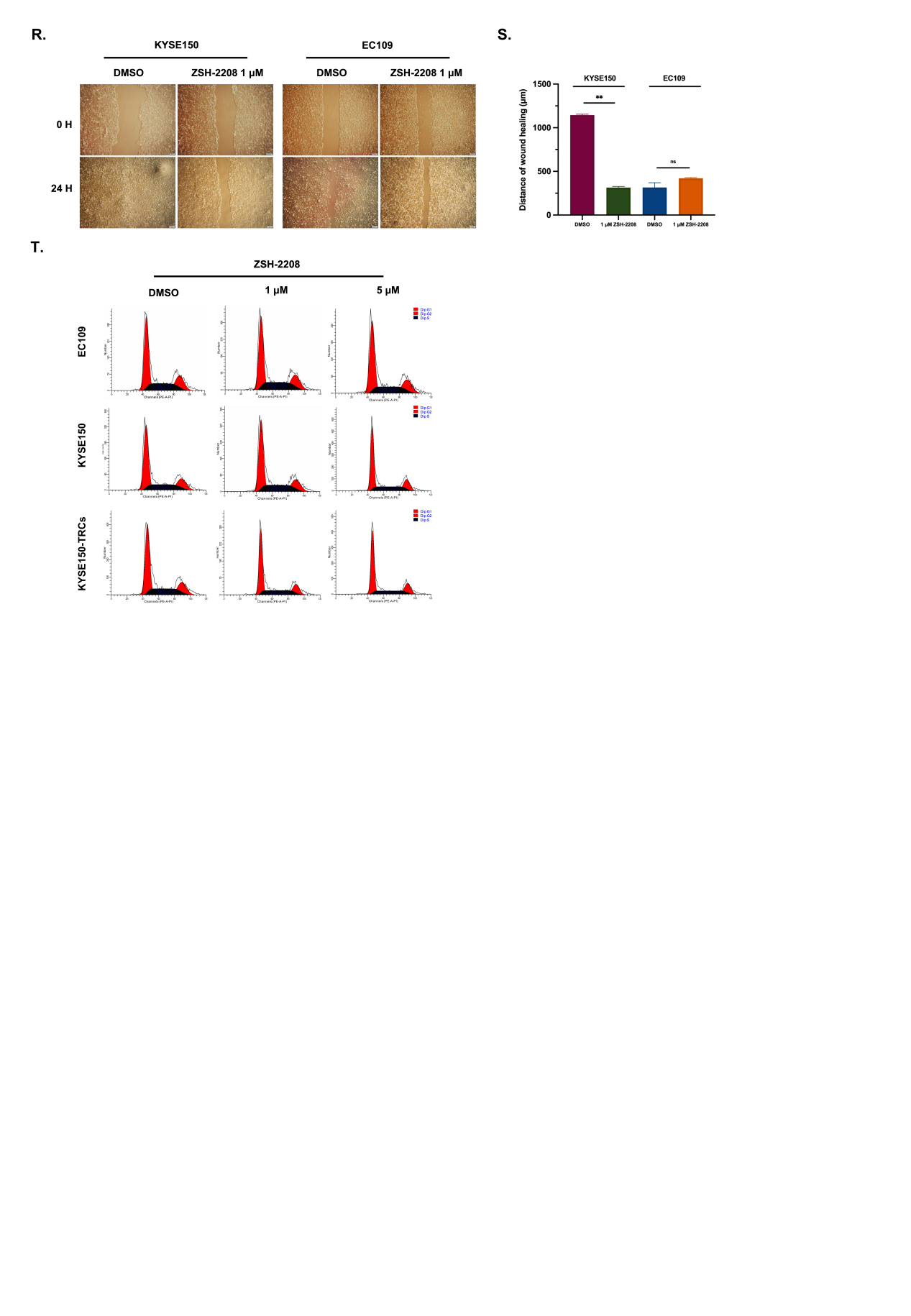
**

**Supplementary Figure 3.** A,B,C,D, ZSH-2208 significantly inhibits the growth of ESCC Cells and ESCC-TRCs. DOX: doxorubicin. E,F,G,H, Effect of ZSH-2208 on apoptosis of ESCC cells. I,J,K, Effect of ZSH-2208 on expression levels of apoptosis marker cleaved-Caspase3 and proliferation marker Ki67 in EC109 and EC109-TRCs. DOX: doxorubicin; cleaved-Caspase3: green; DAPI: blue; Ki67: red. L,M,N,O, Effect of ZSH-2208 on migration of ESCC cells and ESCC-TRCs. P,Q, Effect of ZSH-2208 on invasion of ESCC cells and ESCC-TRCs. R,S, Scratch assays were used to observe the effect of ZSH-2208 on migration of ESCC cells and ESCC-TRCs. T, Effect of ZSH-2208 on cell cycle of ESCC cells and ESCC-TRCs. (*P < 0.05, **P < 0.01, ***P < 0.001, ****P < 0.0001, ns. no significance)

**
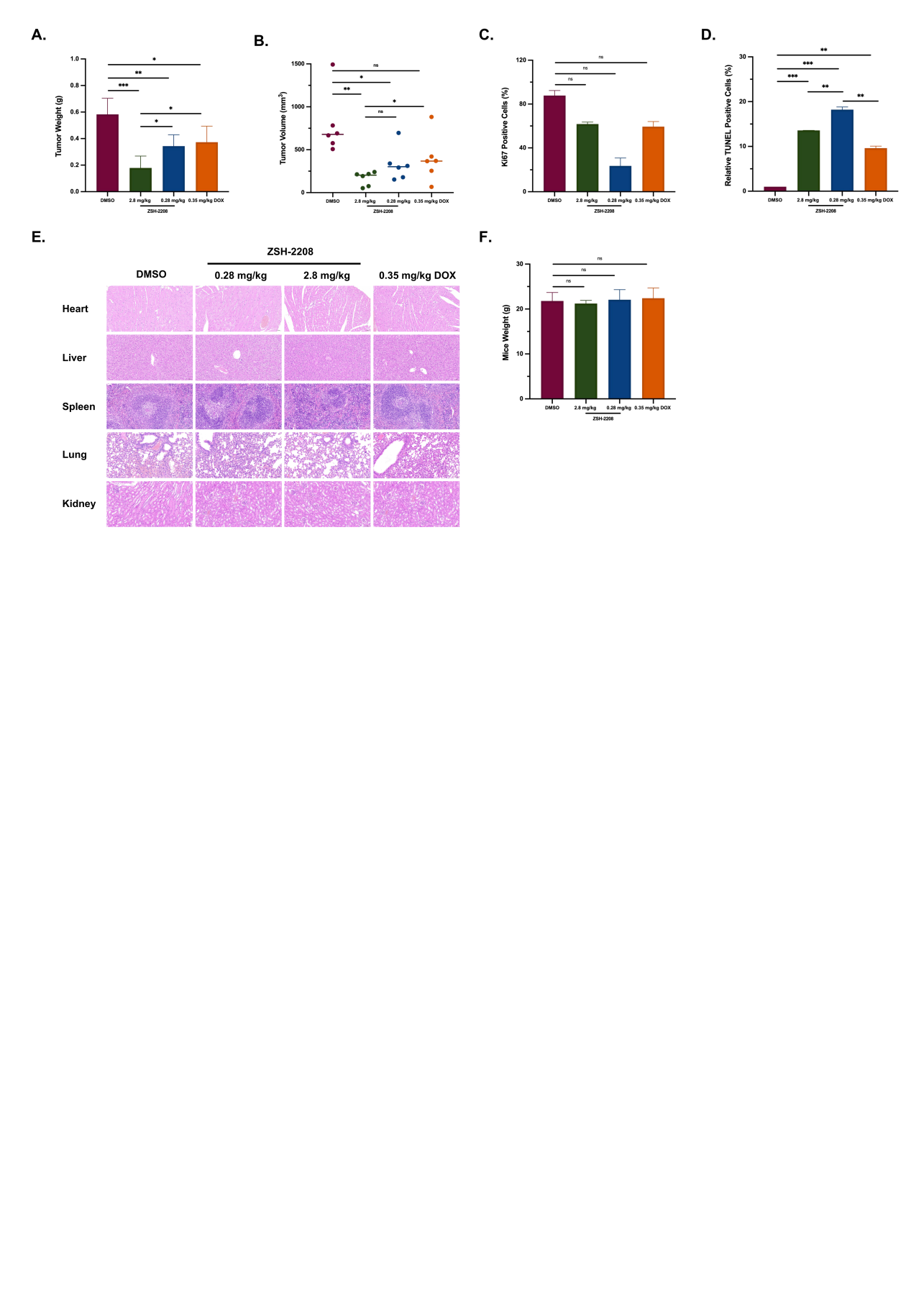
**

**Supplementary Figure 4.** Effect of ZSH-2208 in subcutaneous tumor formation assay in nude mice. And no adverse effects of ZSH-2208 were observed during subcutaneous tumor formation in nude mice.

**
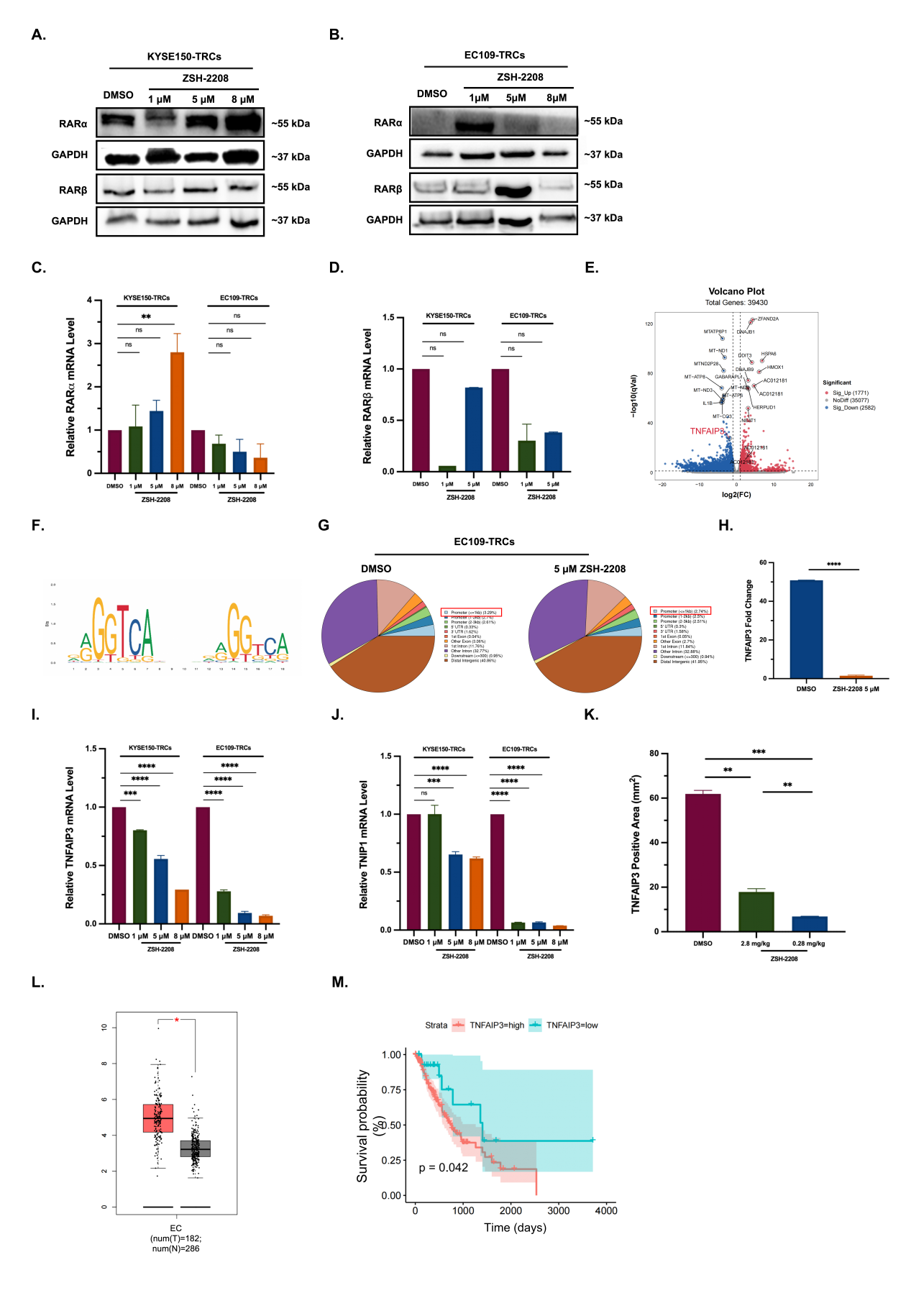
**

**Supplementary Figure 5.** A,B,C,D, Effect of ZSH-2208 on RARs protein and gene expression levels in ESCC-TRCs. E, Volcano plot of the significantly upper (lower) -modulated differentially expressed gene (DEGs) Top50 between ZSH-2208 and DMSO treatment group. F, Motif of TNFAIP3. G. Different regions of genomic functional elements annotated by peaks of RARγ protein account for different regions. H, RARγ protein-enriched gene samples validated by ChIP-PCR. I,J, Effect of ZSH-2208 on TNFAIP3 and TNIP1 at gene level in ESCC-TRCs. K, Effect of ZSH-2208 on TNFAIP3 protein expression in subcutaneous tumor tissues of nude mice. L, The expression of TNFAlP3 in cancerous tissues compared with adjacent non-cancerous tissues in EC patients. M, High TNFAIP3 expression is positively correlated with poor survival in ESCC patients. (*P < 0.05, **P < 0.01, ***P < 0.001, ****P < 0.0001, ns. no significance)

**Supplementary Figure 6.** A,B,C,D TNFAIP3 knockdown EC109 stable transplants were constructed with siRNA1 and siRNA2. E,F Effect of knockdown of TNFAIP3 on migration and invasion of EC109-TRCs and EC109. G,H,I Effect of knockdown of TNFAIP3 on apoptosis in EC109-TRCs and EC109. J, Effect of overexpression of TNFAIP3 on the growth of EC109-TRCs. K, The promoting effect of TNFAIP3 overexpression on clonal sphere formation in KYSE150-TRCs was reversed by ZSH-2208. L,M, Effect of overexpression of TNFAIP3 on the migration and invasion of EC109-TRCs. (*P < 0.05, **P < 0.01, ***P < 0.001, ****P < 0.0001, ns. no significance)
