# Supplementary material for: ZSH‐2208: A novel retinoid with potent anti‐tumour effects on ESCC stem cells via RARγ–TNFAIP3 axis
Source: Clin Transl Med. 2024 Dec 26;15(1):e70148. doi: 10.1002/ctm2.70148 (PMC11670471; doi:10.1002/ctm2.70148)
Supplement: Supplementary file 2 — Supporting information [file CTM2-15-e70148-s001.docx]

**Supplementary Materials**

**Materials and methods**

**Cell Culture and Animal**

The ESCC cell lines KYSE150, EC109, and TE1, procured from the Cell Bank at the Chinese Academy of Sciences (Shanghai, China), were employed for both *in vitro* and *in vivo* experiments. These cells were cultured in RPMI 1640 Medium (Gibco, USA) supplemented with 10% fetal bovine serum (FBS, Gibco, USA) and 1% antibiotics (penicillin/streptomycin 100 U/mL). All cell cultures were maintained in a 37 °C incubator with 5% CO_2_.

Male BALB/c nude mice, weighing 16-20 g and aged 4-6 weeks, were obtained from SLAC Laboratory Animal Limited Liability Company (Shanghai, China). These mice were housed in specific pathogen-free (SPF) conditions. Ethical approval for all animal experiments was granted by the Ethics Committee of Zhongshan Hospital, Fudan University. Every effort was undertaken to minimize suffering and distress experienced by the experimental animals.

**Quantitative Real-Time PCR (qRT-PCR)**

Total RNA was extracted from cells utilizing Trizol® reagent (Life Technologies). The concentration and quality of the obtained total RNA were assessed through NanoDrop. Subsequently, reverse transcription was carried out employing the PrimeScript® RT Reagent Kit with gDNA Eraser (Takara). Quantitative real-time PCR was performed utilizing TB Green Premix Ex Taq II (Takara) in conjunction with the Applied Biosystems 7500 Fast Real-Time PCR System. The two-step PCR amplification comprised pre-degeneration at 95 °C for 30 s (for a single cycle), followed by PCR reactions conducted at 95 °C for 5 s and 60 °C for 34s (for 40 cycles). The CT value was acquired based on the reaction curve subsequent to PCR completion. GAPDH was designated as the reference gene, and the ΔΔCT method was employed for determining the relative expression of the target gene. Specifically, A was calculated as CTTreatment-CTGAPDH, B as CTControl-CTGAPDH, and the fold of differential expression was established as 2-ΔΔCT(A-B). The following PCR primers were utilized:

GAPDH forward primer 5′-GGAAGCTTGTCATCAATGGAAATC-3′ and reverse primer 5′-TGATGACCCTTTTGGCTCCC-3′;

CR-1 forward primer 5′- AGAGGGACGAGCTTCGACC-3′ and reverse primer 5′-TCAGGACGGCATTCGTACTTT-3′;

CD44 forward primer 5′-GAAAGGAGCAGCACTTCAGGA-3′ and reverse primer 5′-TTCTTGCCTCTTGGTTGCTGT-3′;

RARα forward primer 5′-AAGCCCGAGTGCTCTGAGA-3′ and reverse primer 5′-TTCGTAGTGTATTTGCCCAGC-3′;

RARβ forward primer 5′-CCCCAGAACAAGACACCATGA-3′ and reverse primer 5′-CCCCAGAACAAGACACCATGA-3′;

RARγ forward primer 5′-AAAACTGTATCATCAACAAGG-3′ and reverse primer 5′- CTTCACCTCTTTCTTCTTCTTG -3′;

RXRα forward primer 5′-TCTTGAGCAATGCCAGCAG-3′ and reverse primer 5′-CCACAGCTCACACATCCAATC-3′;

TNFAIP3 forward primer 5′-TGCACACTGTGTTTCATCGAC-3′ and reverse primer 5′-ACGCTGTGGGACTGACTTTC-3′;

TNIP1 forward primer 5′-CAGAATGAGTTGCTGAAACA-3′ and reverse primer 5′-TCTCCTCATCTTTGAATGCT-3′.

**Western Blot**

The Western blot procedures, as described elsewhere^[20]^, were adhered to. After washing cells with PBS, they were lysed using RIPA lysis buffer supplemented with 100x protease inhibitors, 100x phosphatase inhibitors, and 0.1 M PMSF (Abcam, USA). Subsequently, cell lysates were harvested via centrifugation at 14000 rpm for 5 minutes at 4 °C. Protein extracts from the supernatant were collected in new tubes, and protein concentration was quantified using the BCA protein assay method. Protein analysis was conducted through 10% SDS-PAGE gel electrophoresis, followed by transfer onto a PVDF membrane. The membrane was subjected to blocking with 5% skim milk in TBST at room temperature for 1 hour. Thorough washing with TBST occurred thrice, each time for 15 minutes. Primary antibody solution (1:1000 dilution) was applied to the membrane, which was then incubated at 4 °C overnight Subsequently, a secondary antibody was employed for 3 hours at room temperature. The immune-reactive signals were visualized using Super Signal West Pico Chemiluminescent Substrate, employing G-BOX chemi-XRQ. Detection signals were quantified using Genesys software. GAPDH (Abcam, cat#[ab181602](https://www.abcam.cn/products/primary-antibodies/gapdh-antibody-epr16891-loading-control-ab181602.html), USA) served as the loading control for normalization purposes, ensuring accurate quantification of the detected signals. Primary antibodies used are: Anti-Retinoic Acid Receptor alpha (Abcam, cat#ab275745, USA), Anti-Retinoic Acid Receptor beta Antibody (Abcam, cat#ab124701, USA), Anti-Retinoic Acid Receptor gamma Antibody (CST, cat#8965T, USA), Anti-TNFAIP3 Antibody ( Abcam, cat#ab92324, USA).

**Cell Proliferation Assay**

Cell viability was assessed employing the Cell Counting Kit-8 (CCK-8, Dojindo Molecular Technologies, Kumamoto, Japan) in line with the manufacturer's guidelines. A 100 µL suspension of EC was seeded at a density of 5x10^4^ cells/mL in 96-well plates and left to incubate overnight. The subsequent day, fresh media supplemented with 10% FBS was introduced. Cells were subjected to treatment, including ATRA, Fluorouracil, ATRA plus Fluorouracil, or no treatment control, for durations of 24 and 48 hours. Following the treatment period, 10 µL of CCK-8 solution was added to each well, allowing an incubation period of one hour. Optical density (OD) values were gauged at 450 nm utilizing the Spectrophotometer NANADROP2000 (Thermo Scientific, USA). The calculation of cell viability was executed using the following formula:

Cell Viability [%] = [(Abs (sample) - Abs (blank)) / (Abs (Negative control) - Abs (blank))] x 100.

**Clonesphere Formation Assay**

Adhering to the guideline of 5000 TRCs per well, cells were introduced into 96-well plates containing 3D soft fibrin gel. Following a 24-hour incubation period, cells were subjected to treatment with either ZSH-2208 or doxorubicin. Over the subsequent 1 to 5 days, the size of the clone spheres was observed and meticulously recorded utilizing an inverted microscope. For each assessment, three distinct fields were chosen for observation. Utilizing the ImageJ software, the diameter of each clone sphere was analyzed. Subsequently, the volume of the clone sphere was computed following the equation V=4/3πR^3^, where "V" represents the volume and "R" symbolizes the radius of the clone sphere.

This assay enabled the quantification of the effects of ZSH-2208 and doxorubicin on clone sphere formation, providing insight into their impact on the growth dynamics of the TRCs.

**Clone Formation Assays**

For the clone formation assays, cells were initially seeded in 6-well plates and allowed to grow for a duration of two weeks or longer. Following this incubation period, cells were fixed using 4% paraformaldehyde (DingGuo, Beijing, China) and subsequently stained with 0.5% crystal violet. Colonies exceeding a size threshold of 0.5 mm were then quantified using ImageJ software, in a manner consistent with previously reported methods. This methodology enabled the assessment of colony-forming capacity, shedding light on the proliferative potential of the cells under investigation.

**Cell Migration and Invasion Assay**

Cell migration and invasion were evaluated using Transwell assays. For both ESCC-TRCs and ESCC cells, 5×10^5^ cells were seeded in 6-well plates and cultured for 24 hours. A sterile 200 µL pipette tip was employed to create a defined scratch, and after removal of non-adherent cells by phosphate-buffered saline (PBS, Gibco) rinse, cells were subjected to serum-free RPMI 1640 incubation under various conditions for 24 hours. Images were captured using a digital camera system 24 hours post-scratch initiation. The experiment was triplicated and repeated at least three times.

For the invasion assay, 24-well plates with 6.5 mm inserts (Corning, USA) were utilized. The upper chamber of these inserts contained 12 µL of ice-cold Matrigel TM (BD, USA)-coated 8 μM polycarbonate membranes. A total of 5 × 10^4^ cells were added to the upper chamber in 200 µL of media with 5% FBS, while the lower chamber contained 500 µL of media with 20% FBS. After 24 hours, cells on the lower side of the membrane were fixed with methanol and stained with 1% crystal violet for quantification. This method enabled the assessment of cell migration and invasion capabilities, enhancing our understanding of the cellular responses under various conditions.

**Cell Transfection**

To achieve stable knockdown (sh_TNFAIP3) or overexpression (oe_TNFAIP3) of specific molecular targets, as well as relevant controls (EV), we initiated retroviral packaging and infection. Recombinant lentivirus was generated for this purpose. The lentivirus, along with 10 mg/mL polybrene, was introduced to the cell suspension for co-culture, and the medium was changed after 24 hours. Following co-culture, infected cells were subjected to selection with puromycin over a period of 3 days. The effectiveness of the infection was assessed via Western Blot, enabling verification of the transfection efficiency and successful manipulation of the target molecules.

**Flow Cytometry**

Cells were detached using Stem Pro Accutase Cell Dissociation Reagent (Thermo Fisher Scientific) and subsequently labeled with FITC anti-human CD44 (Biolegend, cat#397517, USA). As control measures, cells were subjected to staining with PE-anti-Mouse IgG (Biolegend, cat#405307, USA) or APC anti-Mouse IgG antibodies (Biolegend, cat#405308, USA). Additionally, cells were co-stained with DAPI to facilitate proper gating. Expression profiles were analyzed using Summit 4.3 software (Dako, Santa Clara, CA). This methodology enabled the quantitative assessment of CD44 expression levels within the examined cell populations.

**Immunohistochemistry (IHC)**

Tumor tissues were fixed, embedded, and sectioned into 5 µm thick slices. Immunohistochemical staining of TNFAIP3 (Abcam, cat#ab92324, USA) and Ki67 (Abcam, cat#ab15580, USA) was carried out following established protocols. Staining outcomes were examined using a light microscope at a magnification of ×200. Stained slides were independently scored by two investigators utilizing immune-reactive scoring systems, providing a comprehensive assessment of staining intensity and distribution within the tissue samples.

**Immunofluorescence (IF)**

Cells were seeded onto 13-mm round glass coverslips in a 24-well plate, with Matrigel, and incubated in a humidified chamber at 37 ℃ with 5% CO_2_ for 12 hours. For lysosome investigations, cells were cultured with mitochondrial dye, lysoView dye, and Hoechst 33342 for 30 minutes. In the case of immunofluorescence studies, cells were rinsed twice with chilled PBS (pH=7.2), fixed in 4% paraformaldehyde on ice for 20 minutes, and subsequently permeabilized with 0.2% Triton X-100 for 10 minutes. Following PBS washes, cells were subjected to blocking with goat serum for 30 minutes, followed by overnight incubation with primary antibodies . Subsequently, cells were treated with Alexa Fluor 488-conjugated goat anti-rabbit IgG (Abcam, cat#ab150077, USA) or Alexa Fluor 568-conjugated goat anti-mouse IgG (Abcam, cat#ab175473, USA) for 2 hours. After further washing steps, DAPI Staining Solution (Abcam, cat#ab228549, USA) was applied for 5 minutes to label nuclei. Following additional washing and blocking with PVF medium, images were captured using a fluorescence microscope or a Zeiss LSM710 confocal microscope (40x oil immersion). Primary antibodies used are: Anti-Ki67 Antibody (Abcam, cat#ab15580, USA), Anti-TNFAIP3 Antibody (Abcam, cat#ab193462, USA), Anti-Cleaved-Caspase (Abcam, cat#ab32042, USA), TUNEL Assay Kit (CST, cat#25879, USA).
